# Histone H1 binding to nucleosome arrays depends on linker DNA length and trajectory

**DOI:** 10.1038/s41594-022-00768-w

**Published:** 2022-05-17

**Authors:** Marco Dombrowski, Maik Engeholm, Christian Dienemann, Svetlana Dodonova, Patrick Cramer

**Affiliations:** 1 Department of Molecular Biology, Max Planck Institute for Multidisciplinary Sciences, Göttingen, Germany; 2 Present Address: Structural and Computational Biology Unit, European Molecular Biology Laboratory, Heidelberg, Germany

**Keywords:** Cryoelectron microscopy, Chromatin

## Abstract

Throughout the genome, nucleosomes often form regular arrays that differ in nucleosome repeat length (NRL), occupancy of linker histone H1 and transcriptional activity. Here, we report cryo-EM structures of human H1-containing tetranucleosome arrays with four physiologically relevant NRLs. The structures show a zig-zag arrangement of nucleosomes, with nucleosomes 1 and 3 forming a stack. H1 binding to stacked nucleosomes depends on the NRL, whereas H1 always binds to the non-stacked nucleosomes 2 and 4. Short NRLs lead to altered trajectories of linker DNA, and these altered trajectories sterically impair H1 binding to the stacked nucleosomes in our structures. As the NRL increases, linker DNA trajectories relax, enabling H1 contacts and binding. Our results provide an explanation for why arrays with short NRLs are depleted of H1 and suited for transcription, whereas arrays with long NRLs show full H1 occupancy and can form transcriptionally silent heterochromatin regions.

## Abstract (web summary)

Cryo-EM structures of human H1-containing tetranucleosome arrays with distinct, physiological nucleosome repeat lengths reveal that nucleosomes assume a zig-zag arrangement and H1 binds to stacked nucleosomes with longer linker DNA.

## Main

Understanding the organization of the genome requires insights into chromatin structure beyond the level of individual nucleosomes^1,2^. Nucleosomes can be arranged along the DNA into locally structured arrays in the nuclei of eukaryotic cells^1,3^. The relative position of neighboring nucleosomes in such arrays is defined by the nucleosome repeat length (NRL). The NRL comprises the 147 bp of DNA within the nucleosome core particle^4^ and the length of linker DNA that connects the nucleosome with a neighboring nucleosome. The NRL is related to the transcriptional state of genomic regions^5,6^. Active genes contain nucleosome arrays with shorter NRLs, whereas longer NRLs are observed in heterochromatin regions that are transcriptionally silent^7,8^.

The NRL in nucleosome arrays is also associated with differences in the amount of associated linker histone H1, which is one of the most abundant proteins in chromatin^9^. Nucleosome arrays with longer NRLs are associated with higher H1 content, as revealed by studies investigating the effects of changes in H1 levels^8,10–12^ and by studies of H1 stoichiometry across several cell types^13^. H1 is not present in genes that are actively transcribed^14,15^. There are 11 H1 variants in mammalian cells that share a central winged-helix domain, which consists of helices α1–α3, loops L1–L3 and a short, two-stranded β-sheet^16–19^. H1 contacts DNA with L1, the amino-terminal part of α2 together with L3, and α3 to stabilize the nucleosome and contribute to chromatin compaction^17–19^.

There is only limited information on the structure of regular nucleosome arrays^20,21^. Early studies of compacted arrays provided evidence for a two-start helix with nucleosomes stacked along the helix axis^22^. Crystal structures of tetranucleosomes with short NRLs of 157 bp or 167 bp (referred to as 4×157 and 4×167, respectively) showed compact zig-zag arrangements of nucleosomes and lacked H1 (refs. ^23,24^). Later cryogenic electron microscopy (cryo-EM) structures of H1-containing arrays containing 12 nucleosomes and NRLs of 177 bp or 187 bp (referred to as 12×177 and 12×187, respectively) adopted a fiber-like arrangement of stacked tetranucleosome units^25^. However, a crystal structure of an H1-containing array with 6 nucleosomes and an NRL of 187 bp (called here 6x187) showed a less compact ladder-like arrangement^26^. In contrast to these in vitro results, electron tomography found no evidence of regular higher-order arrangements of nucleosomes in vivo^27,28^. Moreover, fluorescence imaging revealed that nucleosomes assemble into small clusters, rather than long fibers, in vivo^29,30^.

In summary, despite considerable efforts by the community, the structure of short nucleosome arrays remains poorly understood. It is also unclear how changes in NRL alter the structure of such arrays and how this influences H1 binding. Here we reconstitute tetranucleosome arrays with four physiologically relevant NRLs in the presence of H1 and analyze the resulting structures by cryo-EM. Our data reveal how the length of linker DNA modulates the local three-dimensional structure of these nucleosome arrays and how this influences H1 binding to particular nucleosomes of the arrays. These results have implications for understanding the compaction and transcriptional activity of chromatin.

## Results

### Structural analysis of tetranucleosome arrays

**Fig. 1:**
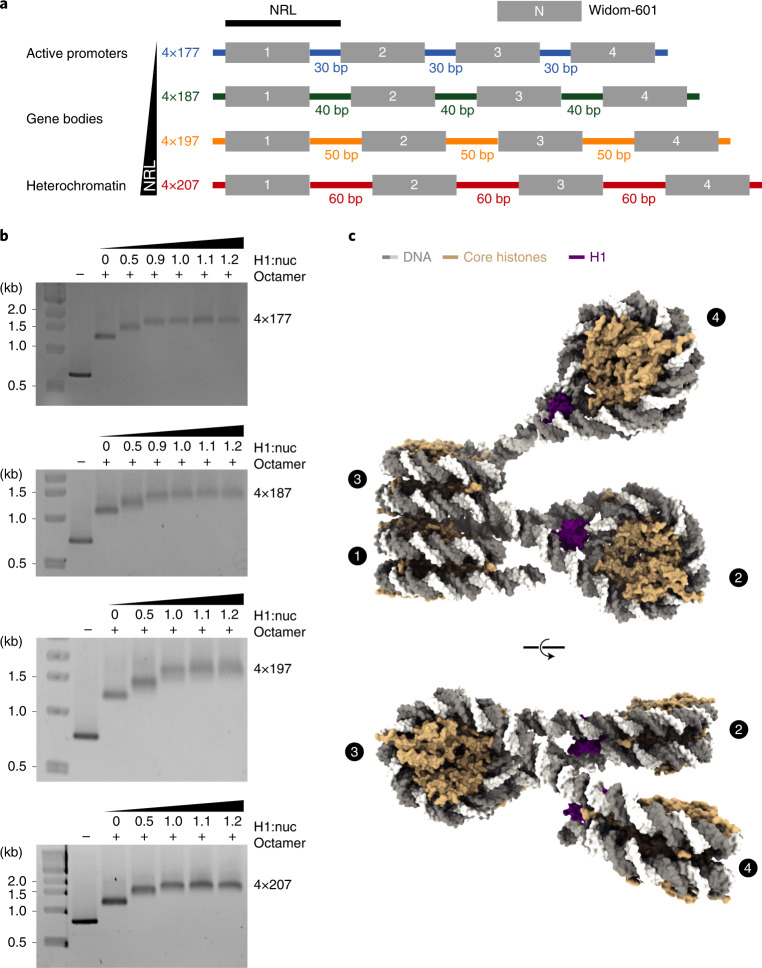
Reconstitution of tetranucleosome arrays for structural studies. **a**, DNA templates contain four Widom-601 (ref. ^56^) nucleosome positioning sequences and variable linker DNA: 4×177 with 30-bp linker, 4×187 with 40-bp linker, 4×197 with 50-bp linker, and 4×207 with 60-bp linker. **b**, EMSA confirms that tetranucleosome arrays were reconstituted with saturating amounts of linker histone H1.4. Stoichiometry of H1 to nucleosome is denoted by H1:nuc. **c**, Structure of the 4×177 tetranucleosome array shows a zig-zag arrangement of nucleosomes, with nucleosomes 1 and 3 forming a stack and nucleosomes 2 and 4 extending from the stack. DNA is shown in gray and white, core histones in wheat, and H1 in purple. Source data

We reconstituted tetranucleosome arrays with four NRLs that occur in human cells in vivo^7^. These NRLs are found near active promoter regions (177 bp), in gene bodies (187 bp, 197 bp) or in heterochromatin (207 bp) (Fig. 1a and Methods). For the reconstitution, we used human histone octamers and saturating amounts of the full-length human linker histone H1 variant H1.4 (Fig. 1b) under previously established conditions^25^. We used restriction enzyme digestion and electrophoretic mobility shift assays (EMSAs) to confirm the integrity of the resulting tetranucleosome arrays that we refer to as 4×177, 4×187, 4×197 and 4×207 (Fig. 1b and Supplementary Fig. 1).

We then used cryo-EM and single-particle analysis to obtain structures of the four tetranucleosome arrays in the absence of NaCl and without crosslinking (Methods). We obtained cryo-EM density maps at 4- to 8-Å resolution and could visualize secondary structure elements in the histone proteins (Supplementary Figs. 2–10). To obtain structural models, we used maps obtained by focused refinement and built individual nucleosome core particles based on an H1.4-bound mononucleosome structure (PDB 7K5Y (ref. ^19^)). The individual nucleosomes adopt a canonical conformation in all our structures^4,31^. Then, we used the overall EM maps to build the linker DNA connecting individual nucleosomes. This resulted in high-quality structures of the four arrays (Tables 1 and 2 and Supplementary Figs. 2–9). We could refine all four nucleosomes in the 4×177 array (Fig. 1c, Supplementary Figs. 2 and 3 and Supplementary Video 1) and could resolve the first three nucleosomes of the 4×187 (Supplementary Figs. 4 and 5 and Supplementary Video 2), 4×197 (Supplementary Figs. 6 and 7 and Supplementary Video 3) and 4×207 arrays (Supplementary Figs. 8 and 9 and Supplementary Video 4).

**Table 1:** Cryo-EM data collection, refinement and validation statistics for the 4×177 and 4×187 arrays

	4×177							4×187					
	nuc 1	nuc 2	nuc 3	nuc 4	stack	trinuc	tetranuc	nuc 1	nuc 2	nuc 3	nuc 4	stack	trinuc
	EMD-13359	EMD-13360	EMD-13361	EMD-13362	EMD-13358	EMD-13357	EMD-13356	EMD-13369	EMD-13368	EMD-13367	EMD-13366	EMD-13365	EMD-13363
	PDB 7PEW	PDB 7PEX	PDB 7PEY	PDB 7PEZ	PDB 7PEV	PDB 7PEU	PDB 7PET	PDB 7PF6	PDB 7PF5	PDB 7PF4	PDB 7PF3	PDB 7PF2	PDB 7PF0
**Data collection and processing**													
Magnification	×81,000	×81,000	×81,000	×81,000	×81,000	×81,000	×81,000	×81,000	×81,000	×81,000	×81,000	×81,000	×81,000
Voltage (kV)	300	300	300	300	300	300	300	300	300	300	300	300	300
Electron exposure (e^−^/Å^2^)	60	60	60	60	60	60	60	60	60	60	60	60	60
Defocus range (μm)	0.5–2.0	0.5–2.0	0.5–2.0	0.5–2.0	0.5–2.0	0.5–2.0	0.5–2.0	0.5–2.0	0.5–2.0	0.5–2.0	0.5–2.0	0.5–2.0	0.5–2.0
Pixel size (Å/pix)	1.05	1.05	1.05	1.05	1.05	1.05	1.05	1.05	1.05	1.05	1.05	1.05	1.05
Symmetry imposed	*C*_1_	*C*_1_	*C*_1_	*C*_1_	*C*_1_	*C*_1_	*C*_1_	*C*_1_	*C*_1_	*C*_1_	*C*_1_	*C*_1_	*C*_1_
Initial particle images	1,341,160	1,341,160	1,341,160	1,341,160	1,341,160	1,341,160	1,341,160	1,259,654	1,259,654	1,259,654	1,259,654	1,259,654	1,259,654
Final particle images	174,476	128,860	174,476	20,621	174,476	174,476	20,621	110,706	61,926	110,706	51,385	110,706	27,515
Map resolution (Å)	4.6	5.1	4.5	7.9	6.0	7.2	9.5	4.0	3.8	4.0	4.0	5.1	11
FSC threshold	0.143	0.143	0.143	0.143	0.143	0.143	0.143	0.143	0.143	0.143	0.143	0.143	0.143
Map resolution range (Å)	4.2–8.0	4.5–7.3	4.1–8.7	7.2–25	4.5–10.8	5.6–11	6.8–25	3.9–11.3	3.4–8.1	3.8–9.2	3.6–9.3	4.0–13.0	8.0–14
**Refinement**													
Initial models used (PDB code)	7K5Y	7K5Y	7K5Y	7K5Y				7K5Y	75KY	75KY	75KY		
Model resolution (Å)	4.6	5.1	4.5	7.9	6.0	7.2	9.5	4.0	3.8	4.0	4.0	5.1	11
Model resolution range (Å)	4.2–8.0	4.5–7.3	4.1–8.7	7.2–25	4.5–10.8	5.6–11	6.8–25	3.9–11.3	3.4–8.1	3.8–9.2	3.6–9.3	4.0–13.0	8.0–14
Map sharpening B factor (Å^2^)	−150	−520	−150	−300	−310	−300	−500	0	−50	0	−50	−50	0
Model composition													
Non-hydrogen atoms	13,304	13, 880	13,099	14,058	26,403	40,119	54,040	13,470	13,470	12,935	13,470	36,610	41,515
Protein residues	768	843	768	843	1,536	2,379	3,222	843	843	768	843	1,611	2,454
DNA	352	354	342	364	694	1040	1396	334	334	334	334	678	1,082
B factors (Å^2^)													
Protein	224	140	252	373	252	203	248	180	152	158	178	217	163
DNA	294	191	315	419	372	302	220	222	190	241	220	325	340
R.m.s. deviations													
Bond lengths (Å)	0.006	0.005	0.006	0.006	0.006	0.006	0.007	0.008	0.006	0.005	0.007	0.007	0.011
Bond angles (°)	0.912	0.932	0.895	0.910	0.957	1.087	1.280	1.054	0.889	0.887	0.944	0.974	1.335
Validation													
MolProbity score	1.3	1.36	1.14	1.46	1.45	1.42	1.64	1.62	1.32	1.41	1.43	1.47	1.56
Clashscore	5.53	6.47	7.45	8.54	8.22	7.70	13.62	9.20	5.86	5.99	6.15	8.76	9.13
Poor rotamers (%)	0.0	0.0	0.0	0.0	0.0	0.0	0.0	0.0	0.0	0.0	0.0	0.0	0.0
Ramachandran plot													
Favored (%)	98.9	98.4	98.4	98.2	98.4	98.6	98.4	97.3	98.2	97.6	97.6	98.2	97.7
Allowed (%)	1.1	1.6	1.6	1.8	1.6	1.4	1.6	2.7	1.8	2.4	2.4	1.8	2.3
Disallowed (%)	0.0	0.0	0.0	0.0	0.0	0.0	0.0	0.0	0.0	0.0	0.0	0.0	0.0

Nuc 1, 2 and 3 refer to nucleosome 1, 2 and 3; stack refers to stacked nucleosomes 1 and 3; trinuc refers to the trinucleosome consisting of nucleosomes 1, 2 and 3; tetranuc refers to the tetranucleosome.

**Table 2:** Cryo-EM data collection, refinement and validation statistics for the 4×197 and 4×207 arrays

	4×197					4×207				
	nuc 1	nuc 2	nuc 3	stack	trinuc	nuc 1	nuc 2	nuc 3	stack	trinuc
	EMD-13372	EMD-13373	EMD-13374	EMD-13371	EMD-13370	EMD-13381	EMD-13382	EMD-13383	EMD-13380	EMD-13379
	PDB 7PFD	PDB 7PFE	PDB 7PFF	PDB 7PFC	PDB 7PFA	PDB 7PFV	PDB 7PFW	PDB 7PFX	PDB 7PFU	PDB 7PFT
**Data collection and processing**										
Magnification	×81,000	×81,000	×81,000	×81,000	×81,000	×81,000	×81,000	×81,000	×81,000	×81,000
Voltage (kV)	300	300	300	300	300	300	300	300	300	300
Electron exposure (e^-^/Å^2^)	60	60	60	60	60	60	60	60	60	60
Defocus range (μm)	0.5–3.0	0.5–3.0	0.5–3.0	0.5–3.0	0.5–3.0	0.5–2.0	0.5–2.0	0.5–2.0	0.5–2.0	0.5–2.0
Pixel size (Å/pix)	1.05	1.05	1.05	1.05	1.05	1.05	1.05	1.05	1.05	1.05
Symmetry imposed	*C*_1_	*C*_1_	*C*_1_	*C*_1_	*C*1	*C*_1_	*C*_1_	*C*_1_	*C*_1_	*C*_1_
Initial particle images	1,075, 418	1,075, 418	1,075, 418	1,075, 418	1,075, 418	1,259,654	1,259,654	1,259,654	1,259,654	1,259,654
Final particle images	113,924	54,212	113,924	113,924	14,348	100,339	41,441	100,339	100,339	18,025
Map resolution (Å)	4.4	4.4	4.3	6.4	9.7	4.4	5.2	4.3	5.0	9.8
FSC threshold	0.143	0.143	0.143	0.143	0.143	0.143	0.143	0.143	0.143	0.143
Map resolution range (Å)	4.0–7.5	3.9–10	3.9–8.5	4.4–12	7–13	4.2–7.4	4.5–9.9	4.1–7.6	4.1–7.6	7–14
**Refinement**										
Initial models used (PDB code)	7K5Y	7K5Y	7K5Y			7K5Y	7K5Y	7K5Y		
Model resolution (Å)	4.4	4.4	4.3	6.4	9.7	4.4	5.2	4.3	5.0	9.8
Model resolution range (Å)	4.0–7.5	3.9–10	3.9–8.5	4.4–12	7–13	4.2–7.4	4.5–9.9	4.1–7.6	4.1–7.6	7–14
Map sharpening B factor (Å^2^)	−50	−100	−50	−50	0	−100	−100	−100	−100	0
Model composition										
Non-hydrogen atoms	13,675	13,880	12,935	26,814	42,335	13,880	13,470	13,880	27,760	44,100
Protein residues	843	843	768	1,611	2,454	843	843	843	1,686	2,529
DNA	334	354	334	688	1,122	354	334	354	708	1,182
B factors (Å^2^)										
Protein	408	202	366	516	324	325	376	330	403	344
DNA	421	252	413	589	391	384	382	380	474	549
R.m.s. deviations										
Bond lengths (Å)	0.006	0.007	0.006	0.006	0.007	0.006	0.006	0.006	0.006	0.006
Bond angles (°)	0.915	0.936	0.921	0.950	1.137	0.864	0.920	0.880	0.894	1.026
Validation										
MolProbity score	1.44	1.39	1.49	1.59	1.50	1.44	1.43	1.39	1.47	1.42
Clashscore	7.78	7.08	9.19	10.58	9.43	8.13	7.86	7.13	8.78	7.69
Poor rotamers (%)	0.0	0.0	0.0	0.0	0.0	0.0	0.0	0.0	0.0	0.0
Ramachandran plot										
Favored (%)	98.0	98.5	98.4	97.8	98.3	98.8	98.9	98.7	98.1	98.7
Allowed (%)	2.0	1.5	1.6	2.2	1.7	1.2	1.1	1.3	1.9	1.3
Disallowed (%)	0.0	0.0	0.0	0.0	0.0	0.0	0.0	0.0	0.0	0.0

### Overall structure of tetranucleosome arrays

**Fig. 2:**
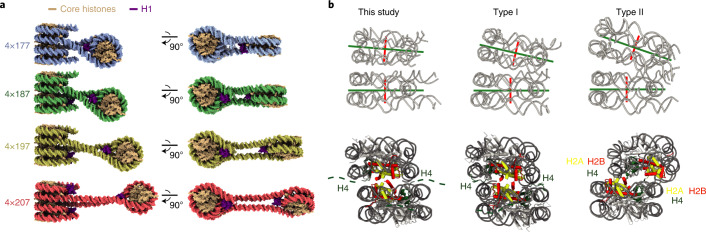
Structure of trinucleosome cores of tetranucleosome arrays. **a**. The trinucleosome cores of the 4×177, 4×187, 4×197 and 4×207 structures. Nucleosome 2 is rotated relative to the stack in all structures and is located at a greater distance from the stack as the length of linker DNA increases. Color code used throughout. **b**, Nucleosome stacking in nucleosome arrays. Nucleosome stacking in tetranucleosome arrays is similar to the stacking observed in the crystal structure of the 4×167 array without H1 (ref. ^23^) and the cryo-EM reconstruction of the 12×177 and 12×187 arrays with H1 (ref. ^25^). Left, nucleosome stack from the 4×187 array represents stacks from all structures reported in this study. Middle: nucleosome stack from the 4×167 crystal structure (PDB 1ZBB (ref. ^23^)) represents the type I interaction observed in the 4×167 crystal structure and within tetranucleosomal units of the 12×177 and 12×187 cryo-EM structures^25^. Right, nucleosome stack from the 6×187 crystal structure (PDB 6HKT (ref. ^26^)) represents the type II interaction observed between tetranucleosome units of the 12×177 and 12×187 cryo-EM structures. Top, dyad axes drawn in green run almost parallel to the stacking observed in the cryo-EM reconstructions determined for 4×177, 4×187, 4×197 and 4×207, whereas dyad axes in the stack observed in both type I and type II interactions are slightly tilted toward each other. Bottom, the interface between stacking nucleosomes in the 4×177, 4×187, 4×197 and 4×207 structures reported here and in type I interactions consists of apposed H2A–H2B dimers (H2A in yellow, H2B in red), while in type II interactions the nucleosome stack is slightly offset and places the N-terminal part of H4 (green) near the H2A–H2B dimer.

All four structures show a zig-zag arrangement of nucleosomes (Fig. 2a), similar to what was observed in the 4×167 array crystal structure without H1 (ref. ^23^) and in designed nucleosome fibers^25,26^. The overall architecture of all tetranucleosome arrays reported here is similar. In all structures, nucleosomes 1 and 3 form a canonical stack^23^, whereas nucleosome 2 is located in a DNA loop between the two stacking nucleosomes and is rotated relative to the nucleosome stack (Fig. 2a). The distance between nucleosome 2 and the nucleosome stack increases with increasing NRL, which leads to increased mobility of nucleosome 2 (Supplementary Figs. 2, 4, 6 and 8). Nucleosome 4 is not stacked with nucleosome 2 and is increasingly mobile as the NRL increases. We were nevertheless able to refine the structure of nucleosome 4 as part of a tetranucleosome in the 4×177 array and also in isolation within the 4×187 array. The linker DNA connecting nucleosomes 3 and 4 was always visible and always showed the same trajectory as in the 4×177 structure.

### Nucleosome stacking in solution

Previous work has revealed two main types of stacking interactions in nucleosome arrays^25,26^. Type I interactions are closely packed stacks with contacts between H2A–H2B dimers, and have been observed in the crystal structure of the 4×167 array without H1 (ref. ^23^) and within the tetranucleosome units of the 12×177 and 12×187 cryo-EM structures^25^ (Fig. 2b). Type II interactions are more open, with slightly offset nucleosomes and the H4 N-terminal tail in close proximity to the acidic patch of the adjacent nucleosome, and have been observed in the 6×187 crystal structure with H1 (ref. ^26^) and between tetranucleosome units of the 12×177 and 12×187 cryo-EM structures^25^ (Fig. 2b). Other stacking interactions have been observed for mononucleosomes in crystals^4^ and by cryo-EM in solution^32^.

In our structures, we observe a compact stacking of nucleosomes 1 and 3 that is similar to type I interactions with a contact formed between H2A–H2B dimers (Fig. 2b). The observed stacking does not allow for interactions between the H4 N-terminal tail of one nucleosome with the acidic patch of a stacked nucleosome and thus leaves the H4 tail free to engage in other interactions^33^. Whereas the inter-nucleosome interactions appear to be very similar, we note a slight relative tilting of the stacking nucleosomes that positions their dyad axes almost parallel, in contrast to type I interactions in which the dyads are slightly tilted toward each other (Fig. 2b). This difference might be due to the absence of H1 in the case of the 4×167 crystal structure^23^ and the different binding mode of H1 to the nucleosome in the case of the 12-mer array with H1 (ref. ^25^).

### H1 orientation and DNA interactions

**Fig. 3:**
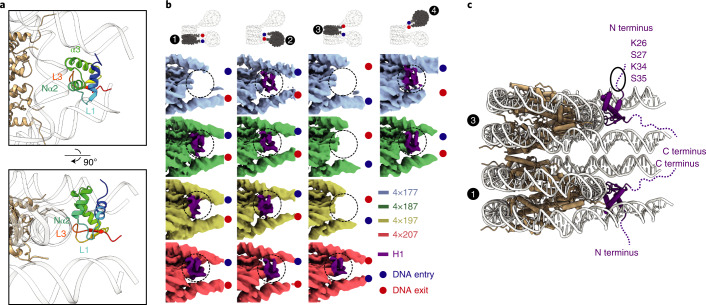
NRL determines H1 binding to arrays. **a**, H1 binds to nucleosomes of the array near the nucleosome dyad. The N-terminal part of the α2-helix (Nα2) and the L3 loop contact the DNA around the dyad, whereas the α3-helix and the L1 loop interact with linker DNAs. H1 is rainbow-colored from the N (blue) to C (red) terminus, DNA is shown in white, and the histone octamer is shown in wheat. **b**, Focused-refined cryo-EM densities for nucleosomes 1, 2, 3 and 4, colored by NRL (4×177 blue, 4×187 green, 4×197 yellow, 4×207 red). H1 density is in purple. Nucleosomes are all viewed the same way. Entry and exit DNA are marked by a blue and a red dot, respectively. Focused-refined maps of nucleosome 4 could not be obtained for the 4×197 and 4×207 arrays owing to higher mobility. **c**. H1 N-terminal regions extend from the nucleosome stack in opposite directions. Residues regulating H1 mobility (K34 (ref. ^34^) and S35 (ref. ^35^)) and heterochromatin formation (K26 and S27)^36^ protrude from the nucleosome stack on both sides and are accessible for protein-protein interactions. The first ordered residue of H1 is S35; disordered residues are shown as a dashed line. DNA is shown in gray, histone octamer in wheat and H1 in purple.

Our structures show that H1 is always bound near the nucleosome dyad (Fig. 3 and Supplementary Figs. 3, 5, 7 and 9). In all ten focused-refined maps, H1 shows three DNA contacts, similar to what has been described^17–19^. The H1 loop L3 and the N-terminal part of helix α2 contact nucleosomal DNA near the dyad, helix α3 binds one linker DNA and loop L1 contacts the other linker DNA (Fig. 3a and Supplementary Figs. 3, 5, 7 and 9). This mode of H1 binding is referred to as on-dyad^17,19^, although H1 is located slightly off the dyad and is lopsided^18^. H1 that is bound to nucleosome 1 always contacts entering linker DNA via its helix α3 (Fig. 3b, Supplementary Figs. 5, 7 and 9 and Supplementary Videos 2–4), whereas H1 on nucleosome 3 uses α3 to contact exiting linker DNA (Fig. 3b, Supplementary Fig. 9 and Supplementary Video 4). In nucleosomes 2 and 4, the entering linker DNA is in contact with α3 (Fig. 3b and Supplementary Figs. 3 and 5; also see Supplementary Video 1).

Thus H1 can be oriented to either contact entering or exiting linker DNA, depending on local DNA geometry. The orientation of H1 influences the direction in which the unstructured N-terminal region of H1 exposes residues to post-translational modifications, such as K34 acetylation, S35 phosphorylation, K26 methylation and S27 phosphorylation^9^ (Fig. 3c). This places N-terminal H1 residues that have been shown to be important for either H1 mobility^34,35^ or heterochromatin formation^36^ at the surface of the nucleosome stack, where they are accessible to modifiers and binding partners even in the presence of a nucleosome stack.

### H1 binding relates to nucleosome repeat length

The major difference between our four structures relates to the binding of the H1 histone to the different nucleosomes of the arrays (Fig. 3b). The H1 histone is present on nucleosome 2 in all four structures, and is also observed on nucleosome 4 in all cases where this nucleosome is structurally resolved. In contrast, the presence of H1 on the stacked nucleosomes 1 and 3 differs between the four arrays. H1 is absent from the stacked nucleosomes of the 4×177 array, but is present on nucleosome 1 in the 4×187 and 4×197 arrays, and is present on both stacked nucleosomes in the 4×207 array. Thus, histone H1 is bound to non-stacked nucleosomes in all structures, whereas H1 binding to stacked nucleosomes is enabled only as the NRL increases.

To confirm that our observations are not a result of low salt concentrations, we solved the trinucleosome core structure of the H1-bound 4×177 array at 60 mM NaCl and confirmed the presence of nucleosome stacks and the absence of H1 on stacking nucleosomes (Supplementary Fig. 11). We have also probed H1 binding to reconstituted 4×177, 4×187, 4×197 and 4×207 arrays biochemically at 150 mM NaCl and observed the that the extent of H1 binding increased with increasing linker length, in line with our structural observations (Supplementary Fig. 12). In conclusion, an increase in NRL is related to stable binding of more H1 copies.

### H1 binding depends on linker DNA trajectory

**Fig. 4:**
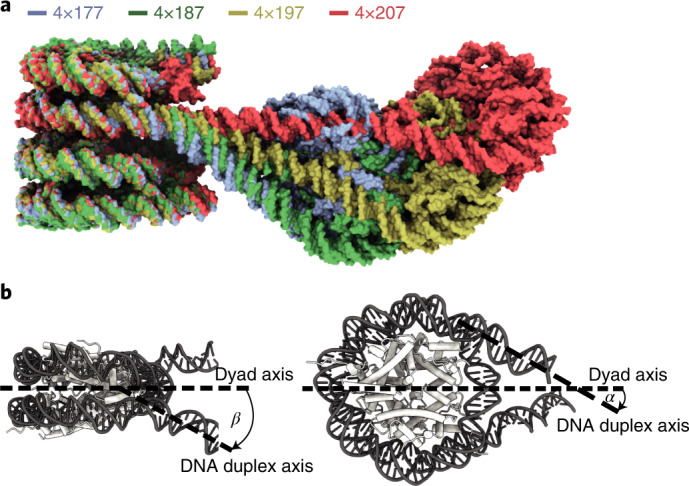
NRL alters linker DNA trajectory at stacked nucleosomes. **a**, Overlay of all four trinucleosome structures shown in Fig. 2. With increasing NRL, linker DNA trajectories at the stacked nucleosomes are altered. **b**, *β* is defined as the angle between the nucleosome dyad and the linker DNA duplex axis, projected onto the plane perpendicular to the nucleosome disc^18^. *α* is defined as the angle between the nucleosome dyad and the linker DNA duplex axis, projected onto the plane of the nucleosome disc^18^.

These observations suggested that linker DNA trajectory determines whether H1 can bind to nucleosomes within an array. We therefore analyzed the linker DNA trajectory at the entry and exit sites of the stacked nucleosomes in all structures. This analysis revealed a progressive change in the trajectory of linker DNA as the NRL increased (Fig. 4). To quantify this, we measured the angles α and β that define linker DNA geometry as described^18^ (Methods and Fig. 4b). Of particular importance here was angle *β*, formed between the nucleosome dyad and the linker DNA duplex axis. We also calculated the differences in angles, Δ*α* and Δ*β*, which are the deviations between the angles α and *β*, respectively, observed in our structures and that in an isolated H1-bound nucleosome (PDB 7K5Y (ref. ^19^)).

**Fig. 5:**
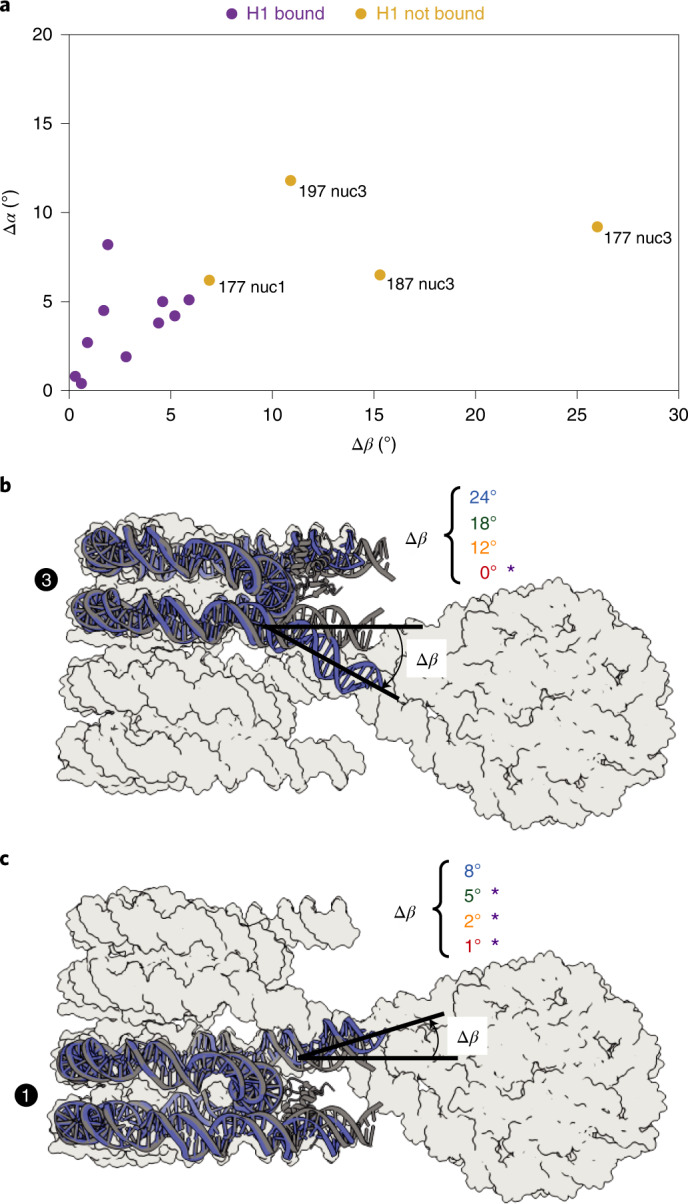
Linker DNA trajectory determines H1 binding. For each nucleosome, Δ*α* and Δ*β* describe the difference in *α* and *β*, respectively, between isolated H1-bound mononucleosomal linker DNA (PDB 7K5Y (ref. ^19^)) and the linker DNA of the nucleosomes in the tetranucleosome array (Supplementary Fig. 1). **a**, A plot of a nucleosome’s average Δ*α* against its average Δ*β* reveals that nucleosomes not bound by H1 (ocher) separate well from the population of nucleosomes bound by H1 (purple). For nucleosome 3, they move closer to this population with increasing NRL. **b**, Δ*β* for nucleosome 3 entry DNA reveals a decrease with increasing NRL. **c**, Δ*β* for nucleosome 1 exit DNA reveals a decrease with increasing NRL. For the depicted nucleosomes, an overlay of the 4×177 nucleosome (blue) and the isolated H1-bound nucleosome (gray) is shown and Δ*β* for the different NRL arrays is listed, with bound H1 indicated by purple asterisks.

**Fig. 6:**
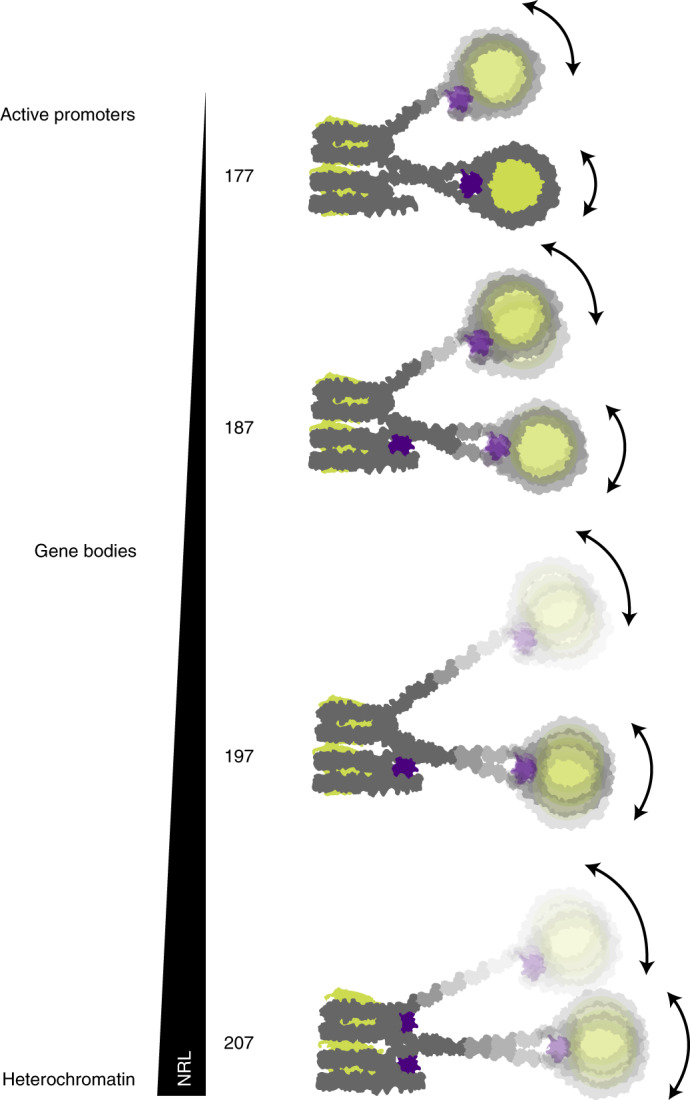
Overview of H1 binding to tetranucleosome arrays. Note that H1 binding to stacked nucleosomes depends on linker DNA trajectory that in turn depends on the NRL. For details, compare text.

Our analysis showed that Δ*β* is a good predictor for histone H1 binding on stacked nucleosomes (Fig. 5). When Δ*β* was close to zero for both linker DNAs emerging from a nucleosome, H1 binding was observed (Fig. 5a). We found low Δ*β* values at nucleosome 2 and Δ*β* values of less than 6° at nucleosome 4, where H1 was always observed (Supplementary Table 1). However, when Δ*β* was higher, H1 was not bound, likely because a stabilizing contact between loop L1 and linker DNA could not be formed. Particularly high Δ*β* values are found for entry DNA at nucleosome 3, except for the 4×207 array, which is the only array where H1 is observed on nucleosome 3 (Fig. 5b). Furthermore, exit DNA of nucleosome 1 shows the highest Δ*β* value for the 4×177 array, which is the only array in which H1 is lacking on this nucleosome (Fig. 5c). In summary, as the NRL increases, nucleosome 2 moves farther away from the stacked nucleosomes and the trajectories of linker DNA at nucleosomes 1 and 3 progressively approach canonical values (Δ*β* = ~0) (Fig. 5a). As a consequence, H1 can contact linker DNA, explaining H1 binding to stacked nucleosomes in arrays with longer NRLs (Fig. 6).

## Discussion

We present cryo-EM structures of tetranucleosome arrays with different NRLs in the presence of the human linker histone H1 variant H1.4. The structures reveal a typical zig-zag arrangement of nucleosomes^23–26^, with a trinucleosome core consisting of two stacked nucleosomes 1 and 3 and a more flexible connecting nucleosome 2, suggesting that a trinucleosome may be a fundamental unit in chromatin^37^. The zig-zag arrangement is observed also in our 4×207 structure, in line with observations from in-cell mapping of DNA contacts^38^. Stacked nucleosomes have also been observed by structural studies of tetranucleosomes, trinucleosomes and free mononucleosomes in solution^32,39–42^. Stacking of nucleosomes 1 and 3 is apparently stabilized by H1 binding to nucleosome 2, because a published structure of a 3×177 trinucleosome array lacking H1 adopts a non-stacked, extended conformation^41^. Our observation of a single nucleosome stack is consistent with small angle X-ray scattering (SAXS) analysis of tetranucleosomes^42^ and hexanucleosome arrays that showed limited compaction^26^. Similar to previous structures of nucleosome arrays^23,25,26^, the structures presented here use NRLs that correspond to those found in vivo^7^ and that differ by integer repeats of the approximate helical repeat of DNA (10n bp linkers with n being a natural number). However, alternative structures of trinucleosomes and tetranucleosomes certainly exist in vivo, and it will be important to study arrays with other linker lengths in the future^43^.

Our major finding here is how the NRL of a nucleosome array relates to H1 binding to the array. It has long been known that there is a correlation between the NRL and the amount of associated H1 (refs. ^12,13^). Additionally, in vitro experiments showed that chromatin with closely spaced nucleosomes does not incorporate H1, whereas chromatin more widely spaced nucleosomes does^44^, but the reasons for this remained elusive. We now report structures that show that short NRLs impair H1 binding (Supplementary Fig. 12) to stacked nucleosomes and suggest this is due to altered linker DNA trajectories. Altered linker DNA trajectories, as observed in our 4×177 array, sterically preclude H1-linker DNA contacts that are required for stable H1 binding^17–19^. A similar observation was made in the structure of a nucleosome containing the H3 variant CENP-A, where an altered linker DNA trajectory has been observed^45^ that precludes H1 binding^41,45,46^. We show that, with increasing NRL, the linker DNA emerging from the stacked nucleosomes is more relaxed and permits stable H1 binding. Therefore, whereas H1 may transiently bind all nucleosomes of the four arrays (Fig. 1b), binding to nucleosomes might be destabilized in short NRL arrays and easily disrupted during cryo-EM sample preparation. We observe canonical on-dyad H1 binding as described^17–19^, in contrast to the off-dyad position of H1 found in tetranucleosome units of 12-mer arrays^25^ that is possibly a result of chemical crosslinking^42^.

Our results have important implications for understanding the relationship between the NRL of a genomic region and its transcriptional activity. In particular, the short NRLs that are characteristic of active promoter regions and transcriptionally active gene bodies^7,8,15^ may preclude H1 from binding to stacked nucleosomes. This could explain the observed depletion of H1 from active promoters^15,47,48^ that likely facilitates assembly of the RNA polymerase II (Pol II) transcription machinery and passage of Pol II through chromatin^14^. The NRL of nucleosome arrays can be defined by chromatin remodeling enzymes^15,49^, and thus remodelers may indirectly deplete H1 by setting short NRLs, thereby complementing other mechanisms of H1 depletion^9,14^ and rendering chromatin permissive to transcription.

Finally, long NRLs are found in heterochromatin regions^7,8,15^, which seems counterintuitive because long NRLs should expose more DNA to the transcription machinery but heterochromatin is transcriptionally silent. Our findings settle this apparent contradiction. We find that longer NRLs are required to enable H1 binding to all nucleosomes of an array, thereby stabilizing nucleosomes and inhibiting chromatin remodeler activity^19,50^. Binding of H1 in turn widens the nucleosomal footprint against which remodelers move neighboring nucleosomes^51–53^ and thus would increase the NRL. Other H1-dependent mechanisms contribute to heterochromatin formation and transcriptional silencing^9,54,55^. For example, recruitment of DNA methyltransferases can downregulate transcription^54^, and heterochromatin protein 1 (HP1) binds to methylated H1 residue K26 (ref. ^36^) and may bridge H1-bound nucleosome stacks to facilitate heterochromatin formation and explain transcription repression.

## Methods

### Plasmids and DNA preparation

Plasmids contained human core histones, H2B1K, H3.2 and H4 (ref. ^57^). Full-length human linker histone H1.4 (UniProt ID P10412) was codon-optimized for *Escherichia coli* and synthesized by IDT as a gBlock. The DNA sequence for the GyrA intein was as described^58^ and was synthesized by IDT as a gBlock. The DNA construct coding for Smt3-H1.4-GyrA was generated by overlap PCR to include a carboxy-terminal 6×His tag and cloned into LIC1B to include an N-terminal 6×His tag. Plasmids containing EcoRV-flanked repeats of the Widom-601 sequence^56^ with DNA linker lengths of 30 bp, 40 bp, 50 bp and 60 bp were synthesized by GeneArt (Thermo Fisher). Linker sequences were based on the design of the 12×177 array^25^. Full DNA sequences are provided in the supplementary information. For DNA preparation, large cultures of *E. coli* XL1 blue transfected with plasmids containing the Widom-601 repeats were grown and prepared using the NucleoBond PC 10000 kit (Macherey-Nagel) according to the manufacturer’s instructions. Purified plasmids were digested with EcoRV (New England Biolabs) overnight, and the DNA templates containing the tandem Widom-601 repeats were purified by precipitation with PEG-6000 (ref. ^59^).

### Protein purification

Human core histones H2A.1, H2B1K, H3.2 and H4 were purified as previously described^57,60^. Purified proteins were flash-frozen in liquid nitrogen and lyophilized. Histone octamer was reconstituted as described^57,60^. In brief, core histones were resuspended in unfolding buffer (6 M guanidinium hydrochloride, 20 mM HEPES pH 7.5, 10 mM dithiothreitol (DTT)), core histones were mixed at molar ratio 1.2:1.2:1:1, dialyzed 3 times against gel filtration buffer (20 mM HEPES pH 7.5, 1 mM EDTA, 2 M NaCl, 2 mM DTT) and loaded onto a Superdex 200 increase 10/300 GL (GE Healthcare) gel filtration column. Peak fractions containing core histone octamer were collected and directly used for nucleosome reconstitution or were flash-frozen in liquid nitrogen and stored at −80 °C.

Full-length human linker histone H1.4 was purified as described^58^, with minor modifications. Briefly, Smt3-H1.4-GyrA was expressed in *E. coli* Rosetta 2 (DE3) cells and purified by His-Trap 5 ml HP (GE Healthcare). Peak fractions containing full-length Smt3-H1.4-GyrA were cleaved by Ulp1 for 1 hour at room temperature, followed by incubation with 500 mM β-mercaptoethanol for 4 hours at room temperature. The sample was adjusted to 8 M urea by weighing in solid urea, added to 1 L of buffer A (50 mM Tris-HCl pH 9.0, 200 mM NaCl, 8 M urea) and purified using a HiTrap SP 1 ml (GE Healthcare) column. The sample was adjusted to 200 mM HEPES pH 7.5 and run over a His-Trap 1 ml HP (GE Healthcare) column. The flowthrough was dialyzed 2 times against buffer B (20 mM HEPES pH 7.0, 600 mM NaCl), concentrated using Amicon Ultra-4 10 kDa MWCO centrifugal filters (Merck Millipore) and directly used for nucleosome reconstitution or flash-frozen in liquid nitrogen and stored at −80 °C.

### Nucleosome array reconstitution

Nucleosome arrays containing H1.4 were reconstituted by salt-gradient dialysis as described^25^. Briefly, histone octamer and DNA were mixed at a molar ratio of 1:1 with respect to Widom-601 sequences in nucleosome reconstitution buffer A (20 mM HEPES pH 7.0, 2 M NaCl, 1 mM EDTA, 1 mM DTT), transferred into Slide-A-Lyzer MINI Dialysis Units 3,500 MWCO (Thermo Fisher) dialysis cups and gradually dialyzed over 16 hours from nucleosome reconstitution buffer A to nucleosome reconstitution buffer B (20 mM HEPES pH 7.0, 600 mM NaCl, 1 mM EDTA, 1 mM DTT). The sample was recovered and reconstituted with H1.4 in 1.2-fold molar excess over the number of Widom-601 sequences and dialyzed for 6 h from nucleosome reconstitution buffer B to nucleosome reconstitution buffer C (20 mM HEPES pH 7.0, 1 mM EDTA, 1 mM DTT). The sample was recovered and cleared from aggregation by spinning down in a table-top centrifuge at the top speed for 10 min at 4 °C. To probe stoichiometric binding of histone octamer to the Widom-601 nucleosome positioning sequence, nucleosome arrays were reconstituted without H1.4 and analyzed by BanI restriction enzyme digestion. For EMSAs of H1-containing arrays, 300 ng of sample was run on a 1.2% agarose gel in 0.5× TBE buffer for 1.5 hours at 110 V at 4 °C. To test differential binding of H1.4 to arrays of different NRLs, nucleosome arrays were reconstituted in the absence of H1.4 and adjusted to 100 nM DNA and 150 mM NaCl. H1.4 was then added to different molar ratios of H1 to Widom-601 sequence and incubated on ice for 30 min, and binding was probed by EMSA as described above. For sample in buffer with salt, the sample was adjusted to 60 mM NaCl and incubated for 30 min on ice prior to cryo-EM grid preparation.

### Cryo-EM sample preparation and data collection

Quantifoil Cu 300 R 1.2/1.3 holey carbon grids were glow-discharged using a PELCO easiGlow (Ted Pella) for 100 s at 15 mA and 0.4 bar. In a Vitrobot Mark IV (FEI) chamber set to 100% humidity at 16 °C, 2 μl of sample was applied to each side of the grid. Excess liquid was blotted away using blot force 5 for 3 seconds, and the grid was vitrified by plunging into liquid ethane. Data were collected on a Titan Krios 300 kV transmission electron microscope (FEI) equipped with a Gatan Imaging Filter set to 20 eV and a K3 direct electron detector (Gatan). Movies containing 60 frames with a total fluence of 60 e^–^/Å^2^ were collected using SerialEM^61^ at a nominal magnification of ×81,000 and a pixel size of 1.05 Å/pixel with 40° stage tilt.

### Data processing and analysis

Gain normalization, motion correction and CTF estimation of cryo-EM movies were performed using Warp^62^, and particles were picked using an instance of Warp’s neural network retrained on the 4×177 data set. Particles were extracted at 8.4 Å/pixel in RELION 3.1 (refs. ^63,64^) and sorted by 2–3 rounds of two-dimensional classification in cryoSPARC^65^. Particles belonging to classes showing 2 or more nucleosomes were reextracted at 3.15 Å/pixel, and all subsequent processing was done in RELION 3.1.

For the 4×177+H1.4 data set (Supplementary Fig. 2), several rounds of 3D classification yielded particles that were refined to a 7.2-Å resolution map of a 4×177 trinucleosome. From this, 3D classification with a mask around the presumed location of the nucleosome 4 yielded particles that were refined to a 9.5-Å resolution map of the 4×177 tetranucleosome. The signal of the trinucleosome was subtracted from these particles, and the output was refined to the 7.9-Å resolution map of the fourth nucleosome. From the 4×177 trinucleosome map, masked refinements on the nucleosome stack or the connecting nucleosome were signal subtracted for the other nucleosomes and refined to yield the focused-refined maps of nucleosomes 1, 2 and 3.

Similarly, the 4×187 (Supplementary Fig. 4), 4×197 (Supplementary Fig. 6) and 4×207 (Supplementary Fig. 8) cryo-EM data were subjected to several rounds of 3D classification and 3D refinement to yield maps with a defined nucleosome stack and blurred density for the connecting nucleosome. From this map, several more rounds of 3D classification were performed, and the selected particles were refined to the 4×187, 4×197 and 4×207 trinucleosome at 11 Å, 9.7 Å and 9.8 Å resolution, respectively. Particles from the 3D refinement of the stack with less defined connecting nucleosome were extracted, unbinned and further processed using signal subtraction, 3D classifications and masked refinements to yield maps for nucleosomes 1, 2 and 3. For the 4×187 data set, the same strategy was applied to obtain the map for nucleosome 4 but proved unsuccessful for the 4×197+H1.4 and 4×207+H1.4 data sets. The angular distribution of views for each map was plotted using Warp, local resolution and global FSC was determined using RELION, and the directional FSCs were calculated using the 3D FSC server^66^.

### Model building and refinement

The local-resolution-filtered maps were used for model building, except for the 4×177 trinucleosome, 4×177 nucleosome 1, 4×177 nucleosome 2 and 4×177 nucleosome 4, for which the post-processed maps were used. For each data set, the structure of the H1-bound mononucleosome (PDB 7K5Y (ref. ^19^)), with protein and DNA sequences mutated to the ones used in this study, was rigid-body fitted into the density of nucleosomal unit in UCSF Chimera^67^. Protein termini, entry DNA and exit DNA were manually adjusted in COOT^68^, and the resulting structures were real-space refined in PHENIX^69^. The refined nucleosomal units were then rigid-body fitted into corresponding densities of the nucleosome stack, trinucleosome and tetranucleosome, respectively, using UCSF Chimera. In case of the trinucleosome and tetranucleosome structures, the linker DNA was manually built in COOT. The models were real-space refined in PHENIX and were validated using Molprobity^70^ (Tables 1 and 2). Figures were generated using PyMOL (Schrödinger), UCSF Chimera and UCSF ChimeraX^70^.

### Analysis of linker DNA trajectories

The models for the nucleosome stacks were used to measure linker DNA trajectories for nucleosomes 1 and 3, and the models of the focused-refined maps of nucleosomes 2 and 4 were used to measure linker DNA deviation for nucleosomes 2 and 4. The corresponding maps were used to rigid-body fit the structure of the H1-bound 197 bp mononucleosome (PDB 7K5Y (ref. ^19^)). The plane of the nucleosome disc needs to be defined to determine the angle *α*, and a plane normal to the nucleosome disc along the dyad axis needs to be defined to determine the angle *β*. For definition of these planes, we defined 3 points for each nucleosomal unit: (1) the centroid of the coordinates of the central base pair of the 147-bp Widom-601 sequence, (2) the centroid of the coordinates of the base pair 38 bp upstream of point 1 and (3) the centroid of the coordinates of the base pair 39 bp downstream of point 1. Points 2 and 3 are on two different DNA gyres and on the opposite side of the nucleosome dyad. We defined vectors **v** using points 2 and 3 to approximate the normal to the nucleosome disc, and **u** using point 1 and the centroid of points 2 and 3 to approximate the dyad axis. We used **u** and **v** to describe the plane perpendicular to the nucleosome disc. We determined the normal **w** to this plane by taking the normalized cross product of **u** and **v**, and we use **u** and **w** to describe the plane of the nucleosome disc. Linker DNA vectors were defined by using (4) the centroid of coordinates of the base pair 5 bp into the Widom-601 sequence and (5) the centroid of the coordinates of the base pair 10 bp outside of the Widom-601 sequence. For measurement of the angle *β*, as shown in Fig. 6b, we projected linker DNA vectors onto the plane generated by **u** and **v** and calculated the angle between the projected vectors. For the angle *α*, linker DNA vectors were projected onto plane the plane generated by **u** and **w** and we calculated the angle between the projected vectors. Calculations were done in MATLAB R2017a.

### Reporting Summary

Further information on research design is available in the Nature Research Reporting Summary linked to this article.

## Online content

Any methods, additional references, Nature Research reporting summaries, source data, extended data, supplementary information, acknowledgements, peer review information; details of author contributions and competing interests; and statements of data and code availability are available at 10.1038/s41594-022-00768-w.

## Supplementary information

Supplementary Information Supplementary Figures 1–12, Supplementary Table 1, Supplementary Videos 1–4 descriptions, DNA sequences, Supplementary References

Reporting Summary

Supplementary Video 1 Nucleosomes 1 and 3 form a stack, while nucleosome 2 loops out between them and nucleosome 4 extends separately from the stack. H1 binds to non-stacking nucleosome 2 and 4 but not to stacking nucleosomes 1 and 3.

Supplementary Video 2 Nucleosomes 1 and 3 form a stack, while nucleosome 2 loops out between them and nucleosome 4 extends separately from the stack. H1 binds to non-stacking nucleosome 2 and 4 and to stacking nucleosome 1 but not to stacking nucleosome 3.

Supplementary Video 3 Nucleosomes 1 and 3 form a stack, while nucleosome 2 loops out between them. H1 binds to non-stacking nucleosome 2 and to stacking nucleosome 1 but not to stacking nucleosome 3.

Supplementary Video 4 Nucleosomes 1 and 3 form a stack, while nucleosome 2 loops out between them. H1 binds to non-stacking nucleosome 2 and to stacking nucleosome 1 and 3.

Supplementary Data 1 Uncropped EMSAs for Supplementary Fig. 1: a, 4×177; b, 4×187; c, 4×197; d, 4×207

Supplementary Data 2 Uncropped EMSA for Supplementary Fig. 11

Supplementary Data 3 Uncropped EMSAs Supplementary Fig. 12: a, 4×177; b, 4×187; c, 4×197; d, 4×207

## Data Availability

Electron microscopy densities have been deposited in the EM Data Bank with the accession codes EMD-13359 to EMD-13383. The coordinate files have been deposited in the Protein Data Bank with the accession codes 7PEW to 7PFX. See Tables 1 and 2. Source data are provided with this paper.

## Source data

Source Data Fig. 1 Uncropped EMSAs: a, 4×177; b, 4×187; c, 4×197; d, 4×207
