# Supplementary material for: Histone H1 binding to nucleosome arrays depends on linker DNA length and trajectory
Source: Nat Struct Mol Biol. 2022 May 17;29(5):493–501. doi: 10.1038/s41594-022-00768-w (PMC9113941; doi:10.1038/s41594-022-00768-w)
Supplement: Supplementary file 1 — Supplementary Figures 1–12, Supplementary Table 1, Supplementary Videos 1–4 descriptions, DNA sequences, Supplementary References [file 41594_2022_768_MOESM1_ESM.pdf]

nature portfolio

https://doi.org/10.1038/s41594-022-00768-w

Supplementary information

Histone H1 binding to nucleosome arrays depends on linker DNA length and trajectory

In the format provided by the authors and unedited

Supplementary materials

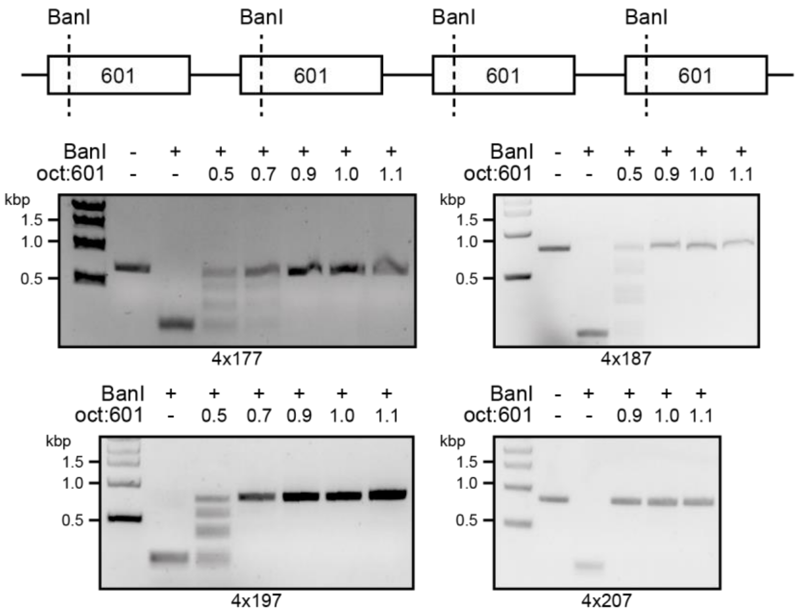

Supplementary Figure 1: Stoichiometric binding of histone octamer to Widom-601 nucleosome positioning sequence. The Widom-601 nucleosome positioning sequence (601) contains a BanI restriction enzyme recognition site at ca. 13 bp. Proper stoichiometry of histone octamer binding to the tetranucleosome DNA template was probed for 4x177, 4x187, 4x197 and 4x207 at different histone octamer to 601 ratios by BanI digest and full protection of BanI sites was observed for all samples at nominally 1:1 stoichiometry, which was then used for subsequent reconstitution of tetranucleosome arrays with H1.

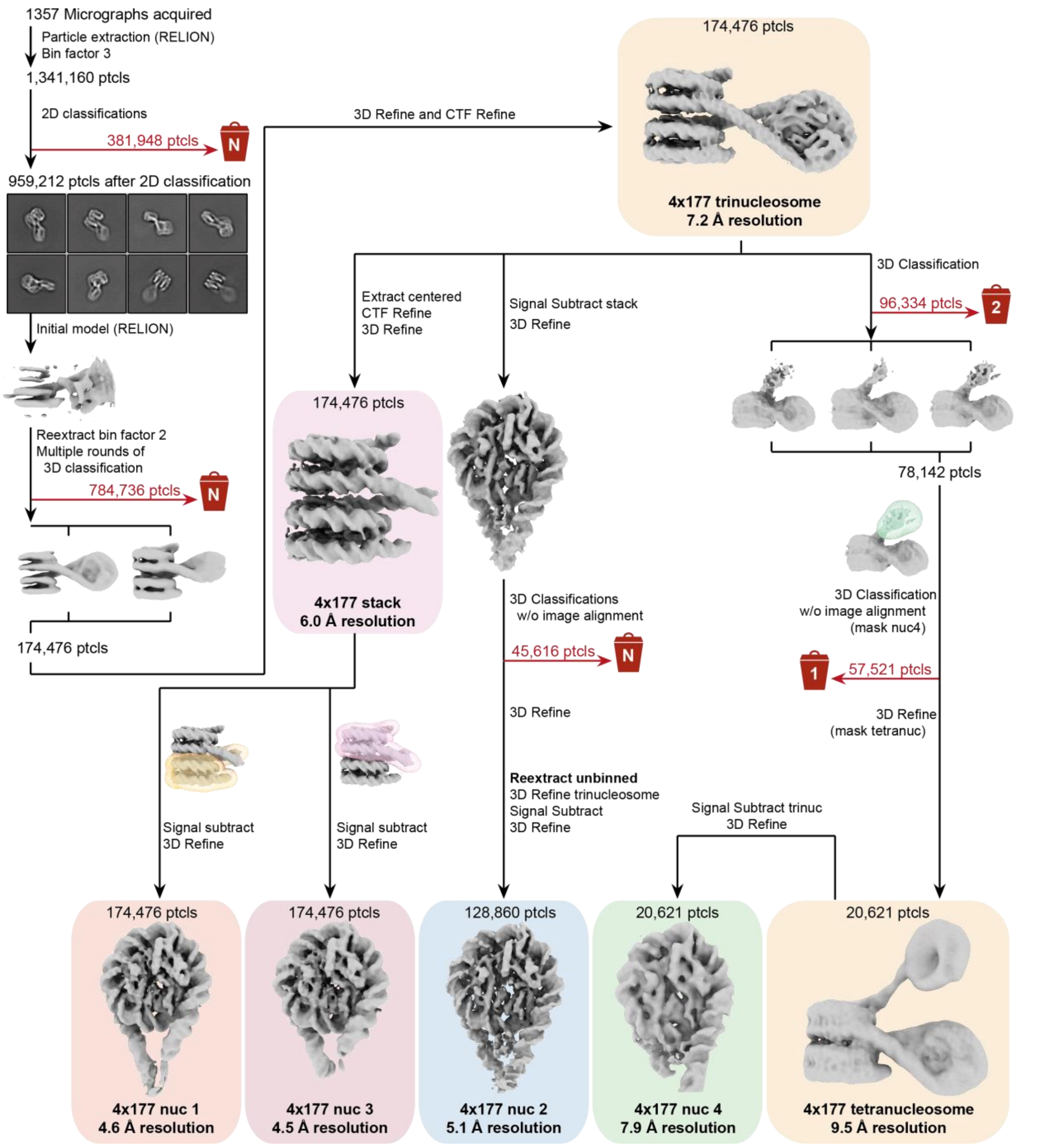

Supplementary Figure 2: Cryo-EM data processing of 4x177 array data set. Particles were picked in Warp[1], extracted 3x binned in RELION[2] and cleaned by several rounds of 2D classification in cryoSPARC[3]. Particles belonging to classes with two or more nucleosomes visible were reextracted 2x binned and used for initial model generation in RELION. Several rounds of 3D classification selecting for trinucleosomes and 3D refinement yielded a trinucleosome map from which signal subtractions and masked classifications and refinements yielded the maps for the individual nucleosomal units and the tetranucleosome. The numbers in the bin denote the number of classes discarded during a classification and is N if more than one classification was performed.

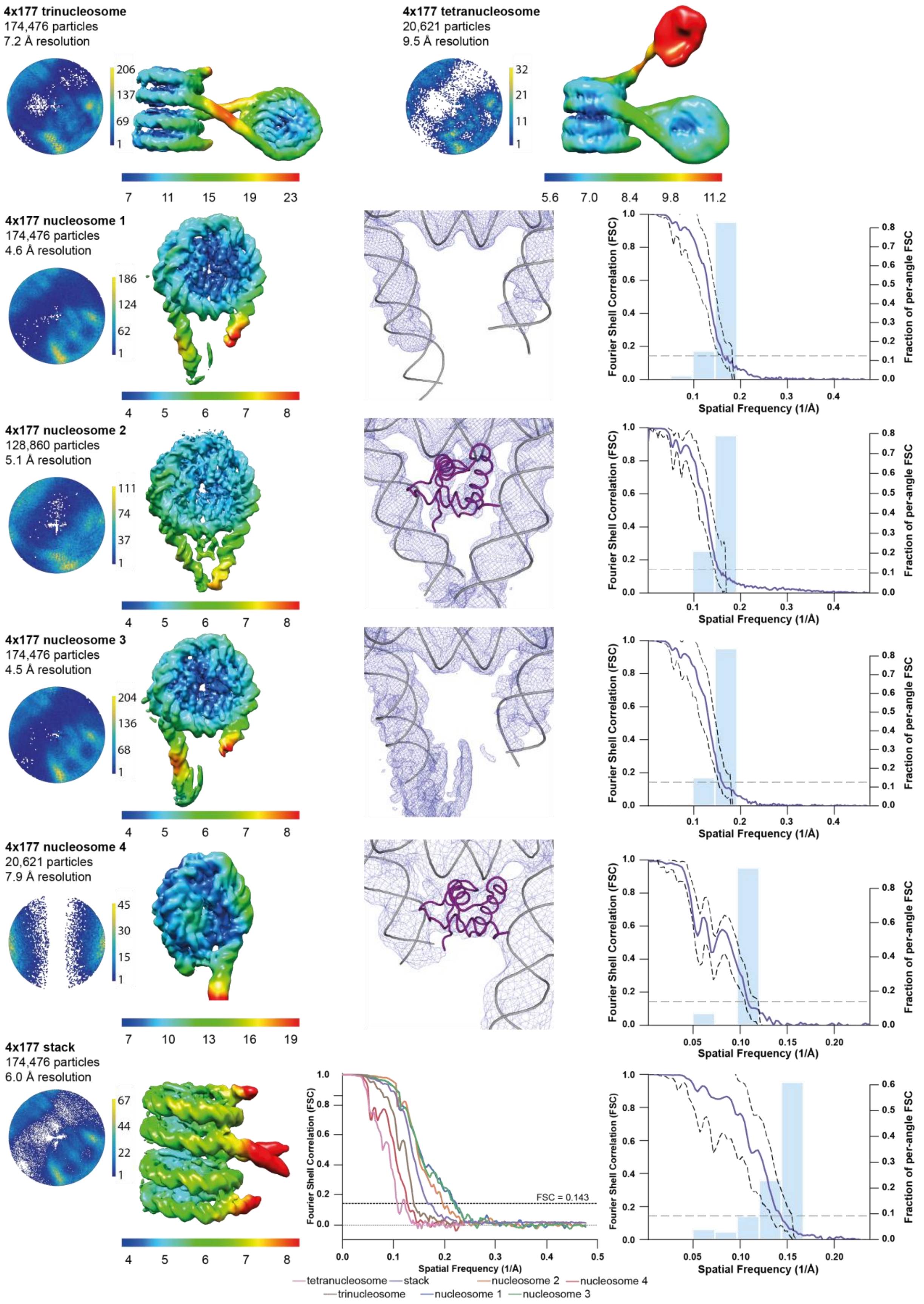
4x177 trinucleosome
174,476 particles
7.2 Å resolution
206
137
69
1
7 11 15 19 23
4x177 tetranucleosome
20,621 particles
9.5 Å resolution
32
21
11
1
5.6 7.0 8.4 9.8 11.2
4x177 nucleosome 1
174,476 particles
4.6 Å resolution
186
124
62
1
4 5 6 7 8
4x177 nucleosome 2
128,860 particles
5.1 Å resolution
111
74
37
1
4 5 6 7 8
4x177 nucleosome 3
174,476 particles
4.5 Å resolution
204
136
68
1
4 5 6 7 8
4x177 nucleosome 4
20,621 particles
7.9 Å resolution
45
30
15
1
7 10 13 16 19
4x177 stack
174,476 particles
6.0 Å resolution
67
44
22
1
4 5 6 7 8
Fourier Shell Correlation (FSC)
Fraction of per-angle FSC
Spatial Frequency (1/Å)
FSC = 0.143
tetranucleosome
stack
nucleosome 2
nucleosome 4
trinucleosome
nucleosome 1
nucleosome 3

Supplementary Figure 3: Angular distribution, local resolution, global FSCs, 3D FSCs and cryo-EM density for the 4x177 array dataset. The cryo-EM reconstructions obtained by the image processing outlined in Supplementary Figure2 are colored by their local resolution determined in RELION[2]. Listed are the map name, number of particles used for the final reconstruction, the nominal resolution determined by RELION using the FSC = 0.143 threshold (FSC plot for all reconstructions bottom row middle panel), and a plot showing angular distribution. For the individual nucleosomal units, the middle panel shows a closeup on the nucleosome dyad with the atomic model (DNA in grey, H1 in purple), and the right panel shows the reconstruction's 3D FSC[4].

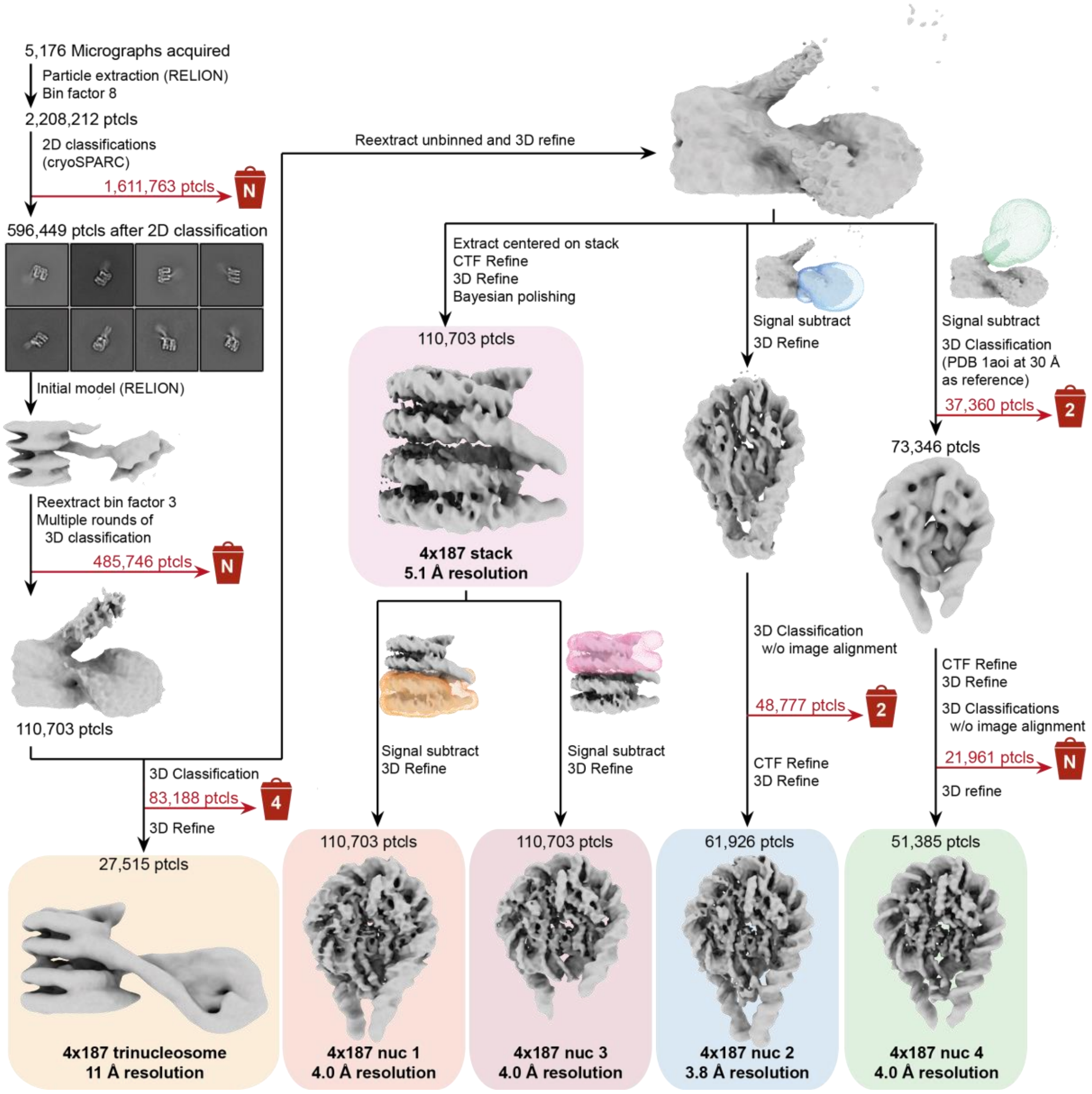

Supplementary Figure 4: Cryo-EM data processing of 4x187 array data set. Particles were picked in Warp[1], extracted 8x binned in RELION[2] and cleaned by several rounds of 2D classification in cryoSPARC[3]. Particles belonging to classes with two or more nucleosomes visible were reextracted 3x binned and used for initial model generation in RELION. Several rounds of 3D classification selecting for trinucleosomes and 3D refinement yielded a trinucleosome map with a defined nucleosome stack and a less defined connecting nucleosome. From this particle set, (a) several rounds of 3D classification and refinement yielded the trinucleosome map and (b) particles were reextracted unbinned, refined and signal subtractions, masked classifications and refinements yielded the maps for the individual nucleosomal units. The numbers in the bin denote the number of classes discarded during a classification and is N if more than one classification was performed.

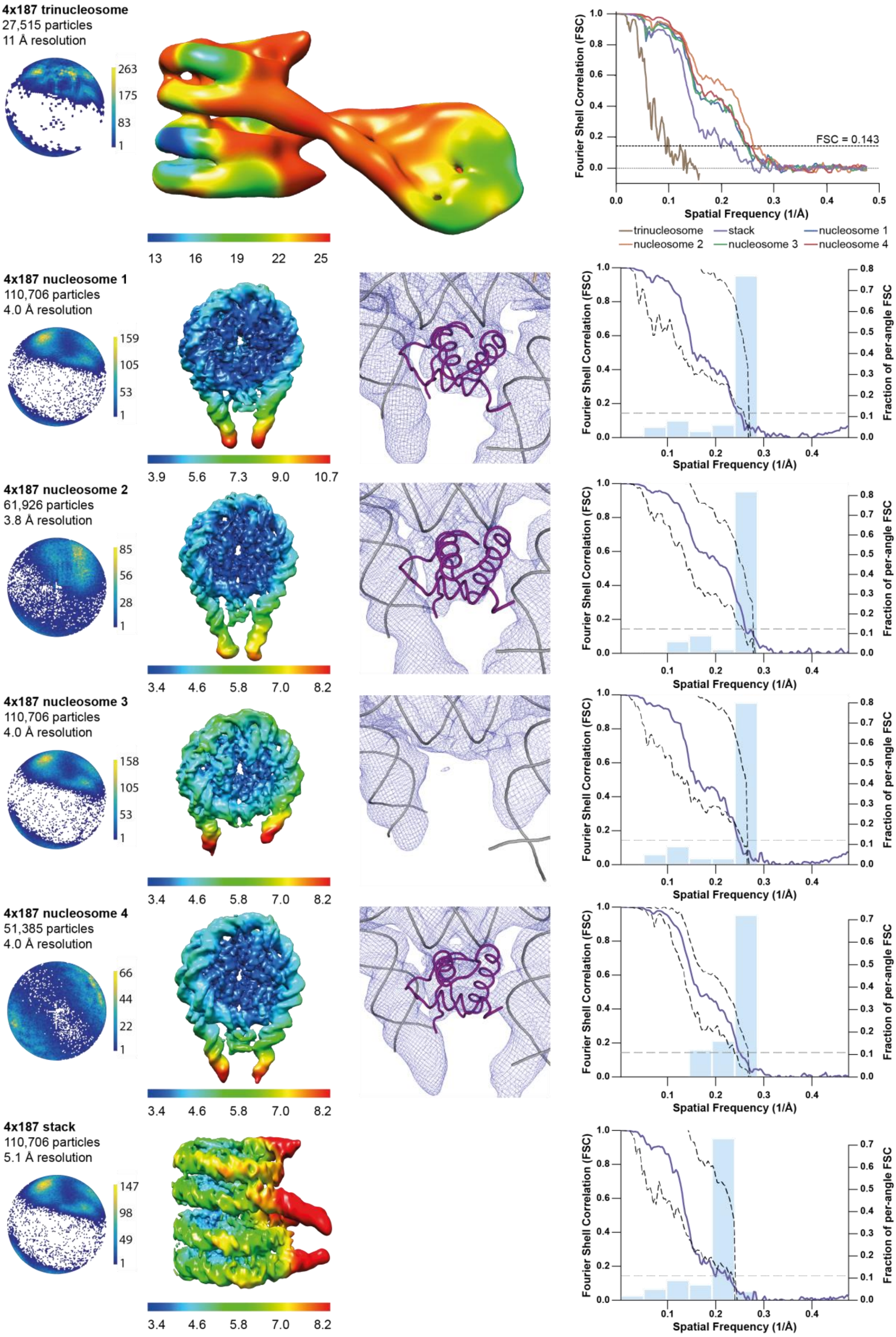

4x187 trinucleosome
27,515 particles
11 Å resolution
263
175
83
1
13 16 19 22 25
Fourier Shell Correlation (FSC)
Spatial Frequency (1/Å)
FSC = 0.143
trinucleosome
stack
nucleosome 1
nucleosome 2
nucleosome 3
nucleosome 4
4x187 nucleosome 1
110,706 particles
4.0 Å resolution
159
105
53
1
3.9 5.6 7.3 9.0 10.7
Fraction of per-angle FSC
4x187 nucleosome 2
61,926 particles
3.8 Å resolution
85
56
28
1
3.4 4.6 5.8 7.0 8.2
4x187 nucleosome 3
110,706 particles
4.0 Å resolution
158
105
53
1
3.4 4.6 5.8 7.0 8.2
4x187 nucleosome 4
51,385 particles
4.0 Å resolution
66
44
22
1
3.4 4.6 5.8 7.0 8.2
4x187 stack
110,706 particles
5.1 Å resolution
147
98
49
1
3.4 4.6 5.8 7.0 8.2

Supplementary Figure 5: Angular distribution, local resolution, global FSCs, 3D FSCs and cryo-EM density for the 4x187 array dataset. The cryo-EM reconstructions obtained by the image processing outlined in Supplementary Figure 4 are colored by their local resolution determined in RELION[2]. Listed are the map name, number of particles used for the final reconstruction, the nominal resolution determined by RELION using the FSC = 0.143 threshold (FSC plot for all reconstructions top row right panel), and a plot showing angular distribution. For the individual nucleosomal units, the middle panel shows a closeup on the nucleosome dyad with the atomic model (DNA in grey, H1 in purple), and the right panel shows the reconstruction's 3D FSC[4].

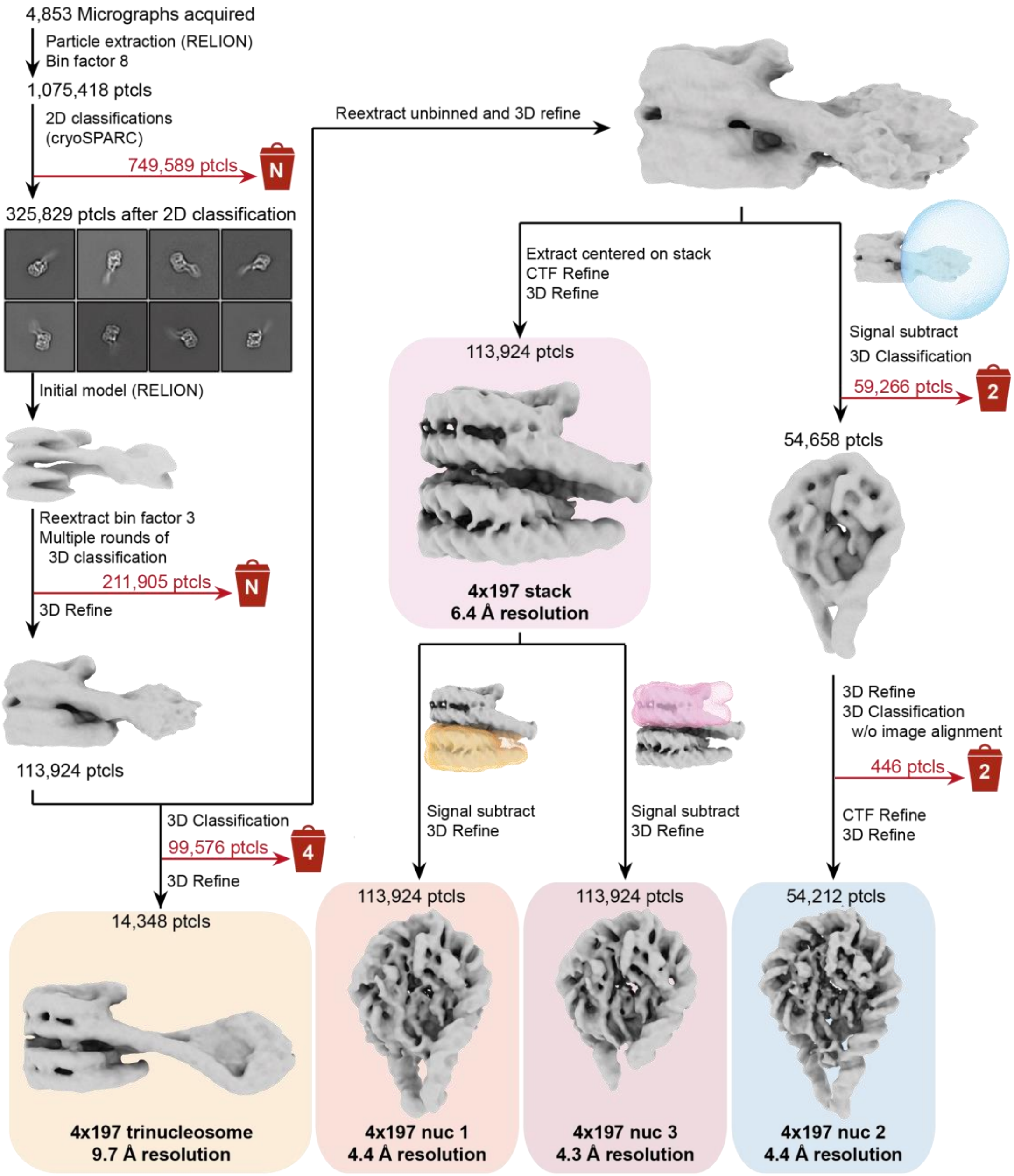

Supplementary Figure 6: Cryo-EM data processing of 4x197 array data set. Particles were picked in Warp[1], extracted 8x binned in RELION[2] and cleaned by several rounds of 2D classification in cryoSPARC[3]. Particles belonging to classes with two or more nucleosomes visible were reextracted 3x binned and used for initial model generation in RELION. Several rounds of 3D classification selecting for trinucleosomes and 3D refinement yielded a trinucleosome map with a defined nucleosome stack and a less defined connecting nucleosome. From this particle set, (a) several rounds of 3D classification and refinement yielded the trinucleosome map and (b) particles were reextracted unbinned, refined and signal subtractions, masked classifications and refinements yielded the maps for the individual nucleosomal units. The numbers in the bin denote the number of classes discarded during a classification and is N if more than one classification was performed.

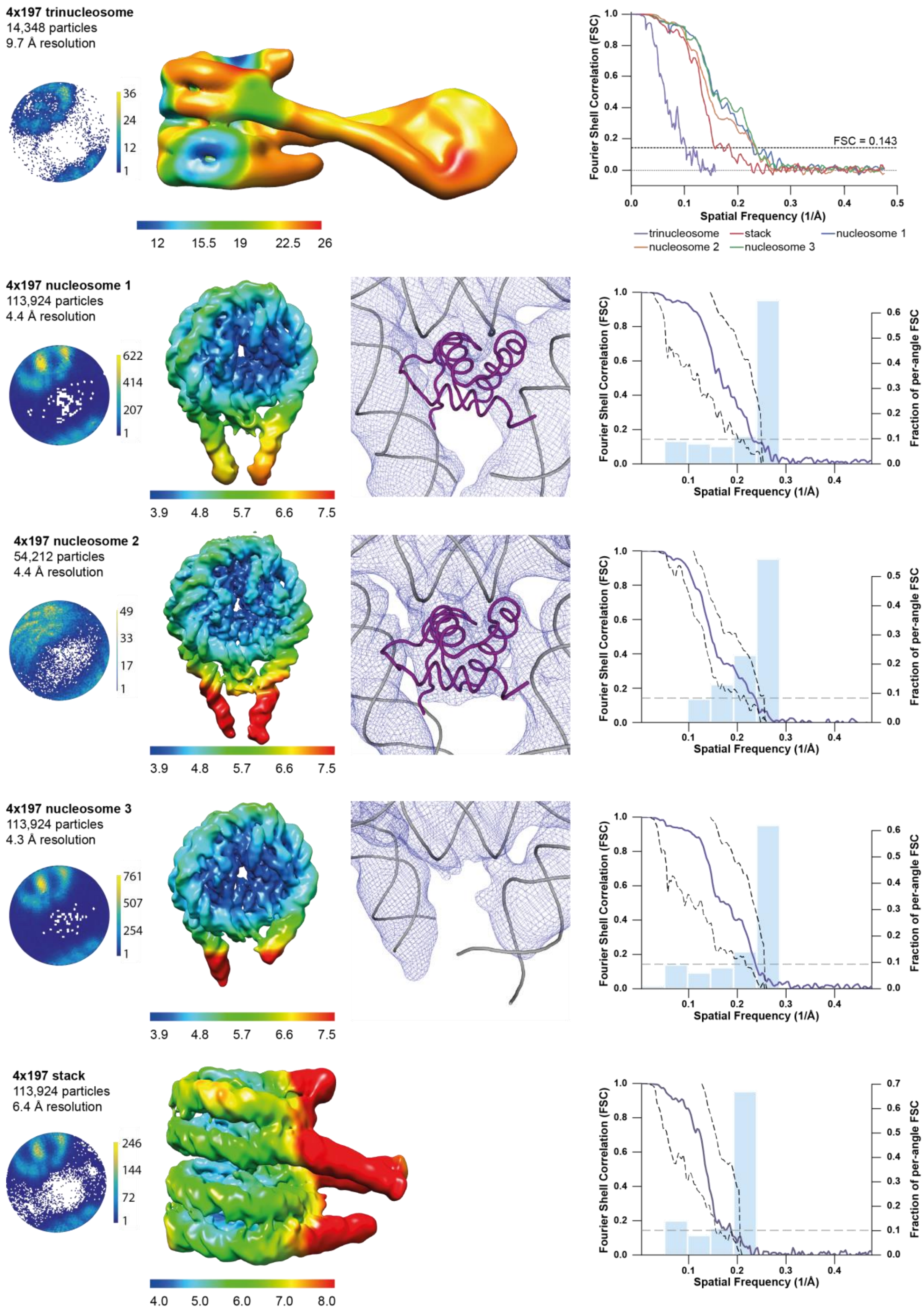
4x197 trinucleosome
14,348 particles
9.7 Å resolution
36
24
12
1
12 15.5 19 22.5 26
Fourier Shell Correlation (FSC)
FSC = 0.143
Spatial Frequency (1/Å)
trinucleosome
stack
nucleosome 1
nucleosome 2
nucleosome 3
4x197 nucleosome 1
113,924 particles
4.4 Å resolution
622
414
207
1
3.9 4.8 5.7 6.6 7.5
Fraction of per-angle FSC
4x197 nucleosome 2
54,212 particles
4.4 Å resolution
49
33
17
1
4x197 nucleosome 3
113,924 particles
4.3 Å resolution
761
507
254
1
4x197 stack
113,924 particles
6.4 Å resolution
246
144
72
1
4.0 5.0 6.0 7.0 8.0

Supplementary Figure 7: Angular distribution, local resolution, global FSCs, 3D FSCs and cryo-EM density for the 4x197 array dataset. The cryo-EM reconstructions obtained by the image processing outlined in Supplementary Figure6 are colored by their local resolution determined in RELION[2]. Listed are the map name, number of particles used for the final reconstruction, the nominal resolution determined by RELION using the FSC = 0.143 threshold (FSC plot for all reconstructions top row right panel), and a plot showing angular distribution. For the individual nucleosomal units, the middle panel shows a closeup on the nucleosome dyad with the atomic model (DNA in grey, H1 in purple), and the right panel shows the reconstruction's 3D FSC[4].

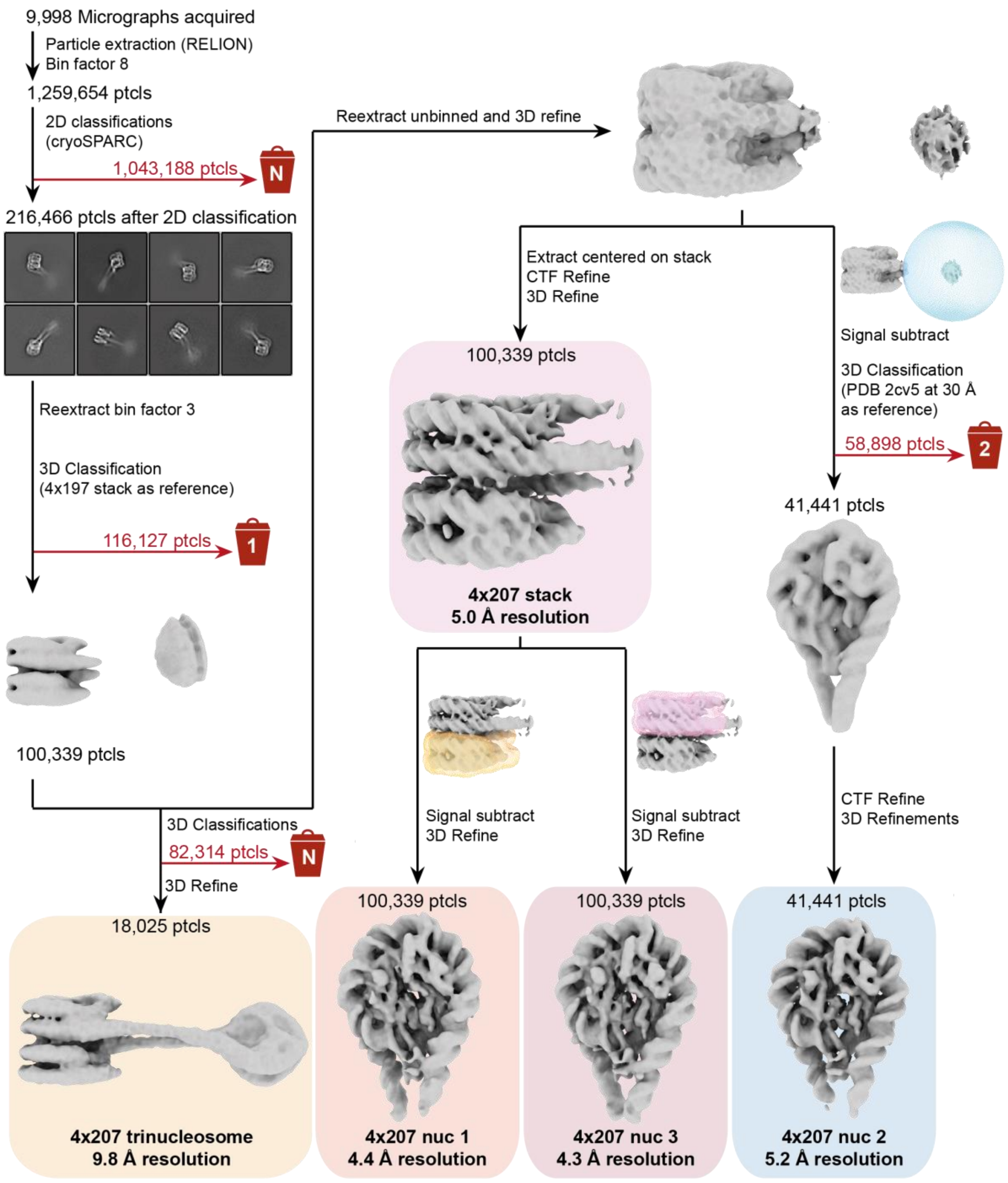

Supplementary Figure 8: Cryo-EM data processing of 4x207 array data set. Particles were picked in Warp[1], extracted 8x binned in RELION[2] and cleaned by several rounds of 2D classification in cryoSPARC[3]. Particles belonging to classes with two or more nucleosomes visible were reextracted 3x binned. Several rounds of 3D classification against the lowpass filtered 4x197 stack map selecting for nucleosome stacks and 3D refinement yielded a trinucleosome map with a defined nucleosome stack and an ill-defined connecting nucleosome. From this particle set, (a) several rounds of 3D classification and refinement yielded the trinucleosome map and (b) particles were reextracted unbinned, refined and signal subtractions, masked classifications and refinements yielded the maps for the individual nucleosomal units. The numbers in the bin denote the number of classes discarded during a classification and is N if more than one classification was performed.

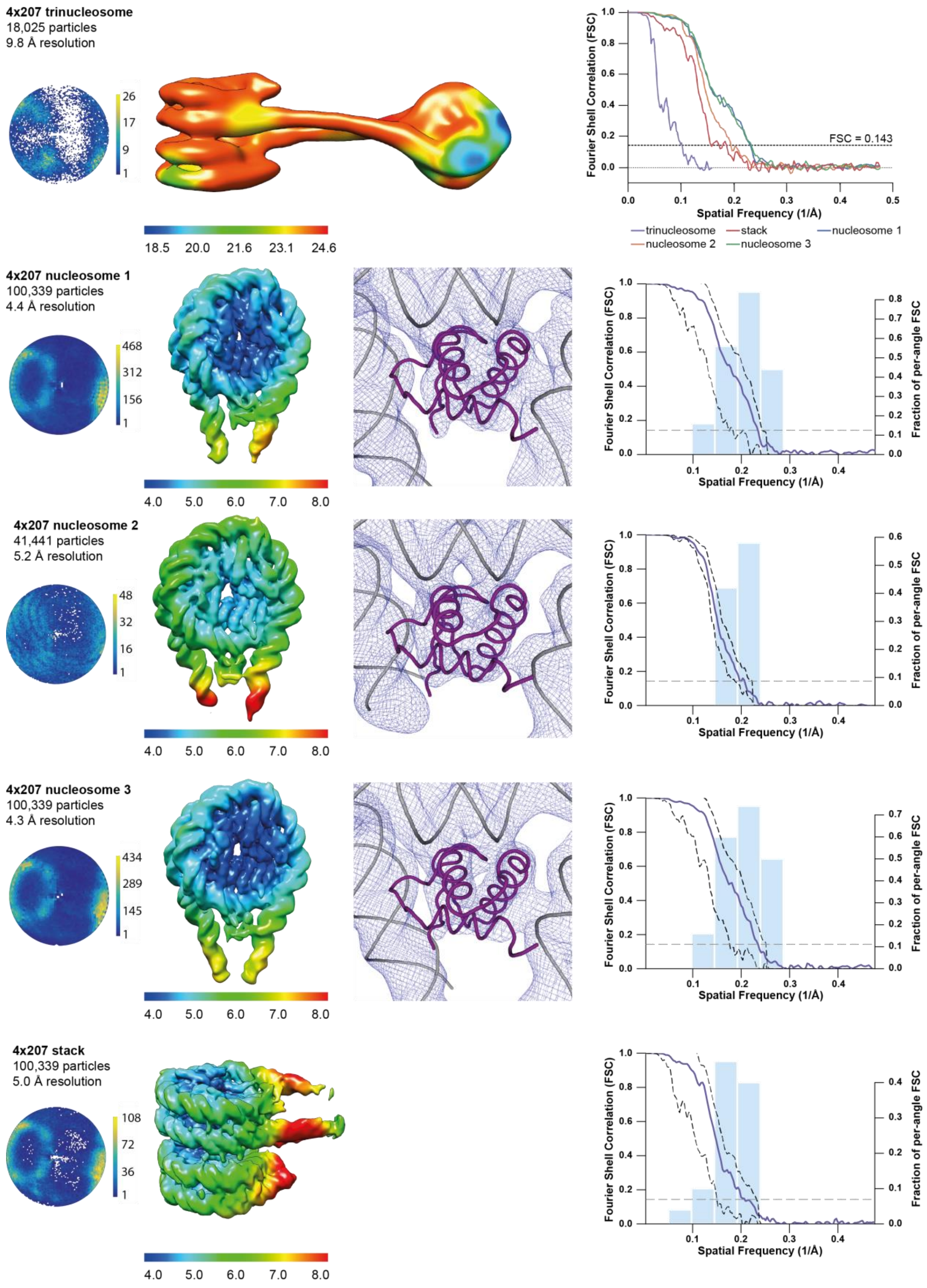
4x207 trinucleosome
18,025 particles
9.8 Å resolution
26
17
9
1
18.5 20.0 21.6 23.1 24.6
Fourier Shell Correlation (FSC)
Spatial Frequency (1/Å)
FSC = 0.143
trinucleosome
stack
nucleosome 1
nucleosome 2
nucleosome 3
4x207 nucleosome 1
100,339 particles
4.4 Å resolution
468
312
156
1
4.0 5.0 6.0 7.0 8.0
Fraction of per-angle FSC
4x207 nucleosome 2
41,441 particles
5.2 Å resolution
48
32
16
1
4.0 5.0 6.0 7.0 8.0
4x207 nucleosome 3
100,339 particles
4.3 Å resolution
434
289
145
1
4.0 5.0 6.0 7.0 8.0
4x207 stack
100,339 particles
5.0 Å resolution
108
72
36
1
4.0 5.0 6.0 7.0 8.0

Supplementary Figure 9: Angular distribution, local resolution, global FSCs, 3D FSCs and cryo-EM density for the 4x207 array dataset. The cryo-EM reconstructions obtained by the image processing outlined in Supplementary Figure8 are colored by their local resolution determined in RELION[2]. Listed are the map name, number of particles used for the final reconstruction, the nominal resolution determined by RELION using the FSC = 0.143 threshold (FSC plot for all reconstructions top row right panel), and a plot showing angular distribution. For the individual nucleosomal units, the middle panel shows a closeup on the nucleosome dyad with the atomic model (DNA in grey, H1 in purple), and the right panel shows the reconstruction's 3D FSC[4].

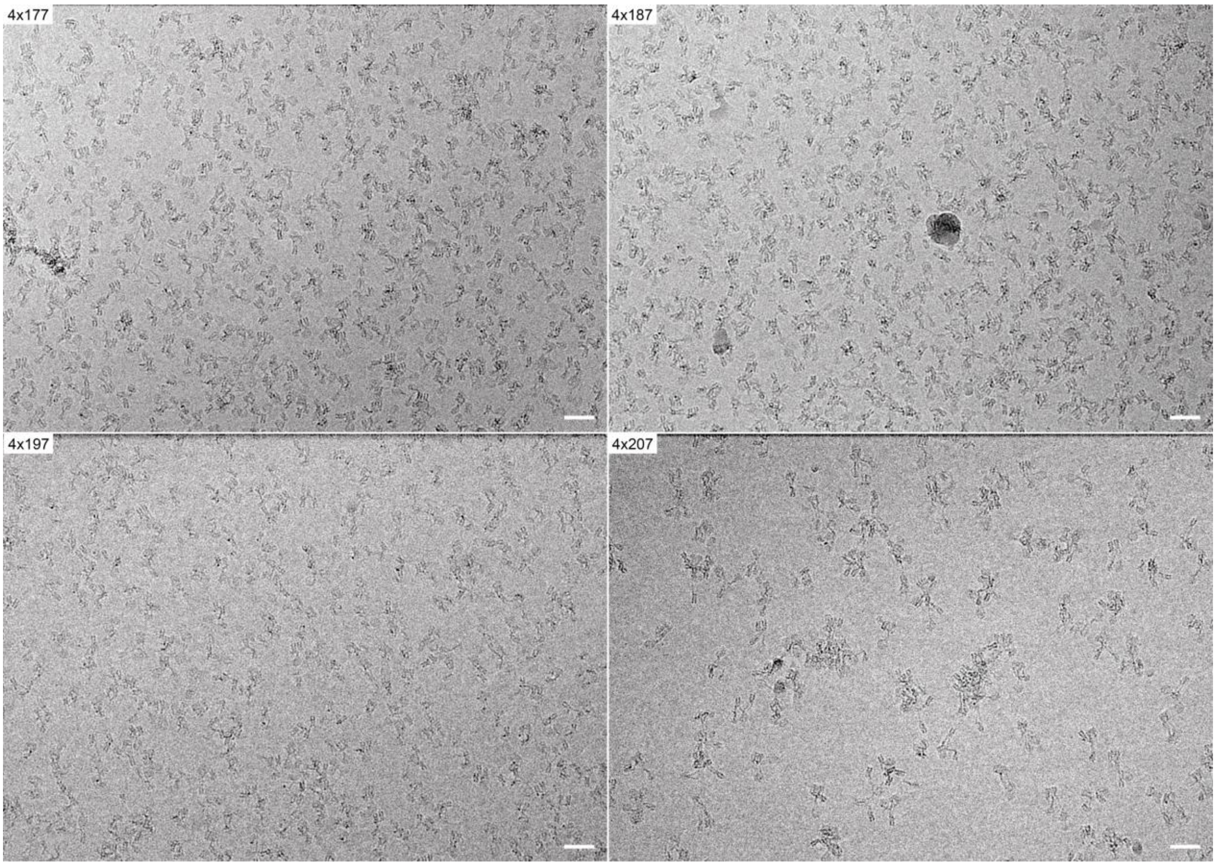

Supplementary Figure 10: Representative cryo-electron micrographs for the 4x177, 4x187, 4x197 and 4x207 data sets. From top left to bottom right: 4x177, 4x187, 4x197, 4x207. Scale bar 30 nm.

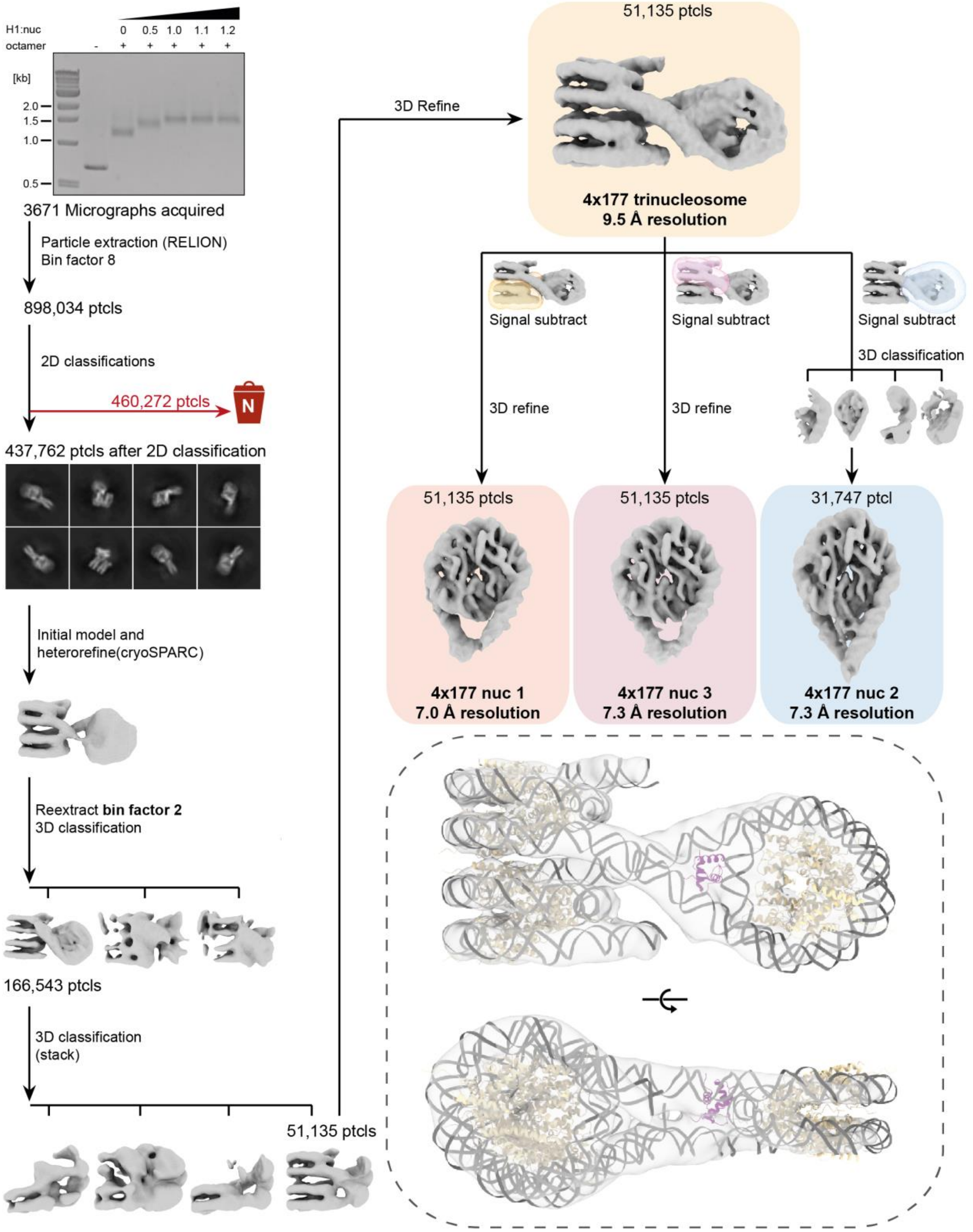

Supplementary Figure 11: Cryo-EM of the 4x177 array in buffer containing 60 mM NaCl at pH 7.5. Inlay bottom right: The 4x177 trinucleosome model at 0 mM NaCl from Supplementary Figure 2 and 3 (DNA in grey, core histones in wheat, H1 in purple) was fit into the 4x177 trinucleosome at 60 mM NaCl cryo-EM reconstruction (transparent grey density).

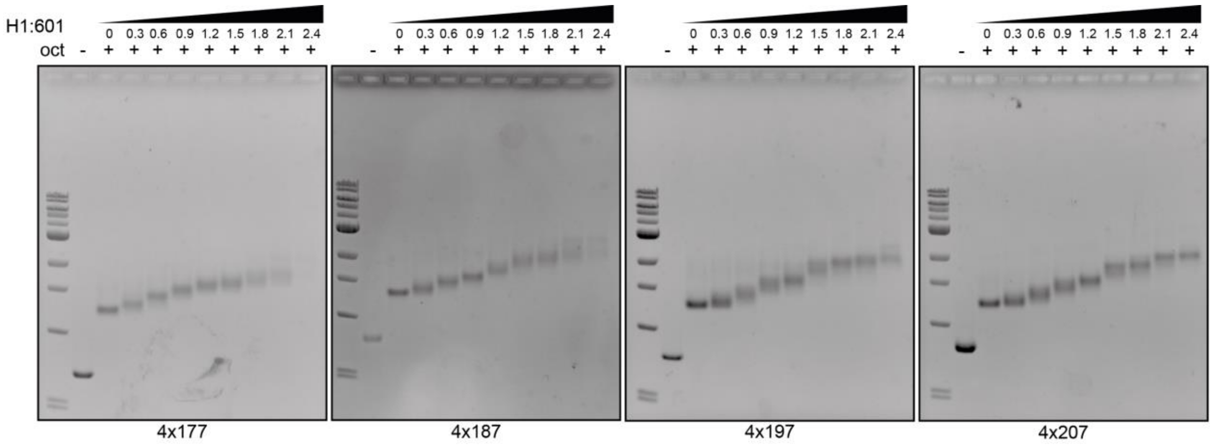

Supplementary Figure 12: H1 binding to different NRL arrays. 4x177, 4x187, 4x197 and 4x207 arrays were reconstituted without H1, adjusted to 100 nM and incubated with different ratios of H1 to histone octamer (Widom-601) at 150 mM NaCl for 30 min on ice. Incorporation of H1 was probed by electrophoretic mobility shift assay and showed an increase in H1 binding capacity with increasing NRL that mimics that observed in the structures. For the 4x177, the shift on the gel stops about midway and progresses with ill-defined bands. For the 4x187 and 4x197, observed shift continues at higher H1 content with increasing band sharpness while for the 4x207 the shift progresses across all assayed H1 ratios and shows distinct bands.

Supplementary Table 1: Exit and entry DNA geometries at nucleosomes in tetranucleosome arrays.

		**nucleosome 1** entry / exit (°)	**nucleosome 2** entry / exit (°)	**nucleosome 3** entry / exit (°)	**nucleosome 4** entry / exit (°)
Δα	**4x177**	9.2 / 3.2	7.8 / 0.4	4.6 / 13.8	2.3 / 0.5
	**4x187**	9.5 / 0.4	0.3 / 9.8	6.9 / 6.0	0.4 / 0.4
	**4x197**	8.8 / 0.2	0.8 / 6.7	3.2 / 18.3	
	**4x207**	9.1 / 8.0	0.8 / 0.7	1.5 / 3.8	
Δβ	**4x177**	5.9 / 7.9	5.4 / 5.0	23.7 / 28.2	5.5 / 0.1
	**4x187**	3.7 / 5.5	6.8 / 5.1	17.7 / 12.8	0.0 / 1.1
	**4x197**	1.6 / 1.8	3.5 / 5.2	12.0 / 8.9	
	**4x207**	3.0 / 0.8	0.3 / 0.2	0.1 / 1.7	

Supplementary Video 1: Cryo-EM structure of the 4x177 array. Nucleosomes 1 and 3 form a stack, while nucleosome 2 loops out between them and nucleosome 4 extends separately from the stack. H1 binds to non-stacking nucleosome 2 and 4 but not to stacking nucleosomes 1 and 3.

Supplementary Video 2: Cryo-EM structure of the 4x187 array. Nucleosomes 1 and 3 form a stack, while nucleosome 2 loops out between them and nucleosome 4 extends separately from the stack. H1 binds to non-stacking nucleosome 2 and 4 and to stacking nucleosome 1 but not to stacking nucleosome 3.

Supplementary Video 3: Cryo-EM structure of the 4x197 array. Nucleosomes 1 and 3 form a stack, while nucleosome 2 loops out between them. H1 binds to non-

stacking nucleosome 2 and to stacking nucleosome 1 but not to stacking nucleosome 3.

Supplementary Video 4: Cryo-EM structure of the 4x207 array. Nucleosomes 1 and 3 form a stack, while nucleosome 2 loops out between them. H1 binds to non-stacking nucleosome 2 and to stacking nucleosome 1 and 3.

DNA sequences

DNA sequences for the 4x177, 4x187, 4x197 and 4x207 arrays are listed below with the Widom-601[5] nucleosome positioning sequences underlined. The DNA sequence for the 4x177 was based on the design of the 12x177 array[6] and after cleavage by EcoRV was:

ATCCCGGATCCCCTGGAGAATCCCGGTGCCGAGGCCGCTCAATTGGTCGTAGA
CAGCTCTAGCACCGCTTAAACGCACGTACGCGCTGTCCCCCGCGTTTTAACCG
CCAAGGGGATTACTCCCTAGTCTCCAGGCACGTGTCACATATATACATCCTGTT
CCAGTGCCGGACCCGAGCATCCGGATCCCCTGGAGAATCCCGGTGCCGAGGC
CGCTCAATTGGTCGTAGACAGCTCTAGCACCGCTTAAACGCACGTACGCGCTGT
CCCCCGCGTTTTAACCGCCAAGGGGATTACTCCCTAGTCTCCAGGCACGTGTC
ACATATATACATCCTGTTCCAGTGCCGGACCCGAGCATCCGGATCCCCTGGAGA
ATCCCGGTGCCGAGGCCGCTCAATTGGTCGTAGACAGCTCTAGCACCGCTTAA
ACGCACGTACGCGCTGTCCCCCGCGTTTTAACCGCCAAGGGGATTACTCCCTA
GTCTCCAGGCACGTGTCACATATATACATCCTGTTCCAGTGCCGGACCCGAGCA
TCCGGATCCCCTGGAGAATCCCGGTGCCGAGGCCGCTCAATTGGTCGTAGACA
GCTCTAGCACCGCTTAAACGCACGTACGCGCTGTCCCCCGCGTTTTAACCGCC
AAGGGGATTACTCCCTAGTCTCCAGGCACGTGTCACATATATACATCCTGTTCC
AGTGCCGAT

The DNA sequence for the 4x187 after cleavage by EcoRV was:

ATCTCTCGCGCACTGGCCGCCTGGAGAATCCCGGTGCCGAGGCCGCTCAATTG
GTCGTAGACAGCTCTAGCACCGCTTAAACGCACGTACGCGCTGTCCCCCGCGT
TTTAACCGCCAAGGGGATTACTCCCTAGTCTCCAGGCACGTGTCAGATATATAC
ATCCTGTCATGTAAGTATTAAGGTAACCCGTCTCGCGCACTGGCCGCCTGGAGA
ATCCCGGTGCCGAGGCCGCTCAATTGGTCGTAGACAGCTCTAGCACCGCTTAA
ACGCACGTACGCGCTGTCCCCCGCGTTTTAACCGCCAAGGGGATTACTCCCTA
GTCTCCAGGCACGTGTCAGATATATACATCCTGTCATGTAAGTATTAAGGTAACC
CGTCTCGCGCACTGGCCGCCTGGAGAATCCCGGTGCCGAGGCCGCTCAATTG
GTCGTAGACAGCTCTAGCACCGCTTAAACGCACGTACGCGCTGTCCCCCGCGT
TTTAACCGCCAAGGGGATTACTCCCTAGTCTCCAGGCACGTGTCAGATATATAC
ATCCTGTCATGTAAGTATTAAGGTAACCCGTCTCGCGCACTGGCCGCCTGGAGA
ATCCCGGTGCCGAGGCCGCTCAATTGGTCGTAGACAGCTCTAGCACCGCTTAA
ACGCACGTACGCGCTGTCCCCCGCGTTTTAACCGCCAAGGGGATTACTCCCTA
GTCTCCAGGCACGTGTCAGATATATACATCCTGTCATGTAAGTATTAAGGTGAT

The DNA sequence for the 4x197 after cleavage by EcoRV was:

ATCGTCTCGCGCACTGGCCGCCATACTGGAGAATCCCGGTGCCGAGGCCGCTC
AATTGGTCGTAGACAGCTCTAGCACCGCTTAAACGCACGTACGCGCTGTCCCC
CGCGTTTTAACCGCCAAGGGGATTACTCCCTAGTCTCCAGGCACGTGTCAGATA
TATACATCCTGTCATGTAAGTATTAAGGTAACCCAGTACTGTCTCGCGCACTGG
CCGCCATACTGGAGAATCCCGGTGCCGAGGCCGCTCAATTGGTCGTAGACAGC
TCTAGCACCGCTTAAACGCACGTACGCGCTGTCCCCCGCGTTTTAACCGCCAA
GGGGATTACTCCCTAGTCTCCAGGCACGTGTCAGATATATACATCCTGTCATGT
AAGTATTAAGGTAACCCAGTACTGTCTCGCGCACTGGCCGCCATACTGGAGAAT

CCCGGTGCCGAGGCCGCTCAATTGGTCGTAGACAGCTCTAGCACCGCTTAAAC
GCACGTACGCGCTGTCCCCCGCGTTTTAACCGCCAAGGGGATTACTCCCTAGT
CTCCAGGCACGTGTCAGATATATACATCCTGTCATGTAAGTATTAAGGTAACCCA
GTACTGTCTCGCGCACTGGCCGCCATACTGGAGAATCCCGGTGCCGAGGCCG
CTCAATTGGTCGTAGACAGCTCTAGCACCGCTTAAACGCACGTACGCGCTGTCC
CCCGCGTTTTAACCGCCAAGGGGATTACTCCCTAGTCTCCAGGCACGTGTCAG
ATATATACATCCTGTCATGTAAGTATTAAGGTAACCCGAT

The DNA sequence for the 4x207 after cleavage by EcoRV was:

ATCCTGGCCGCCACTGGCCGCCACTGGCCACTGGAGAATCCCGGTGCCGAGG
CCGCTCAATTGGTCGTAGACAGCTCTAGCACCGCTTAAACGCACGTACGCGCT
GTCCCCCGCGTTTTAACCGCCAAGGGGATTACTCCCTAGTCTCCAGGCACGTG
TCAGATATATACATCCTGTGCATGTAAGTGCATGTAAGTGCATGTAAGTACTCTG
GCCGCCACTGGCCGCCACTGGCCACTGGAGAATCCCGGTGCCGAGGCCGCTC
AATTGGTCGTAGACAGCTCTAGCACCGCTTAAACGCACGTACGCGCTGTCCCC
CGCGTTTTAACCGCCAAGGGGATTACTCCCTAGTCTCCAGGCACGTGTCAGATA
TATACATCCTGTGCATGTAAGTGCATGTAAGTGCATGTAAGTACTCTGGCCGCC
ACTGGCCGCCACTGGCCACTGGAGAATCCCGGTGCCGAGGCCGCTCAATTGGT
CGTAGACAGCTCTAGCACCGCTTAAACGCACGTACGCGCTGTCCCCCGCGTTT
TAACCGCCAAGGGGATTACTCCCTAGTCTCCAGGCACGTGTCAGATATATACAT
CCTGTGCATGTAAGTGCATGTAAGTGCATGTAAGTACTCTGGCCGCCACTGGCC
GCCACTGGCCACTGGAGAATCCCGGTGCCGAGGCCGCTCAATTGGTCGTAGAC
AGCTCTAGCACCGCTTAAACGCACGTACGCGCTGTCCCCCGCGTTTTAACCGC
CAAGGGGATTACTCCCTAGTCTCCAGGCACGTGTCAGATATATACATCCTGTGC
ATGTAAGTGCATGTAAGTGCATGTAGAT

Supplementary references

1. Tegunov, D. & Cramer, P. Real-time cryo-electron microscopy data preprocessing with Warp. *Nat Methods* **16**, 1146-1152 (2019).
2. Scheres, S.H. RELION: implementation of a Bayesian approach to cryo-EM structure determination. *J Struct Biol* **180**, 519-30 (2012).
3. Punjani, A., Rubinstein, J.L., Fleet, D.J. & Brubaker, M.A. cryoSPARC: algorithms for rapid unsupervised cryo-EM structure determination. *Nat Methods* **14**, 290-296 (2017).
4. Tan, Y.Z. et al. Addressing preferred specimen orientation in single-particle cryo-EM through tilting. *Nat Methods* **14**, 793-796 (2017).
5. Lowary, P.T. & Widom, J. New DNA sequence rules for high affinity binding to histone octamer and sequence-directed nucleosome positioning. *J Mol Biol* **276**, 19-42 (1998).
6. Song, F. et al. Cryo-EM study of the chromatin fiber reveals a double helix twisted by tetranucleosomal units. *Science* **344**, 376-80 (2014).
