# Supplementary figures and images for: Histone H1 binding to nucleosome arrays depends on linker DNA length and trajectory
Source: Nat Struct Mol Biol. 2022 May 17;29(5):493–501. doi: 10.1038/s41594-022-00768-w (PMC9113941; doi:10.1038/s41594-022-00768-w)

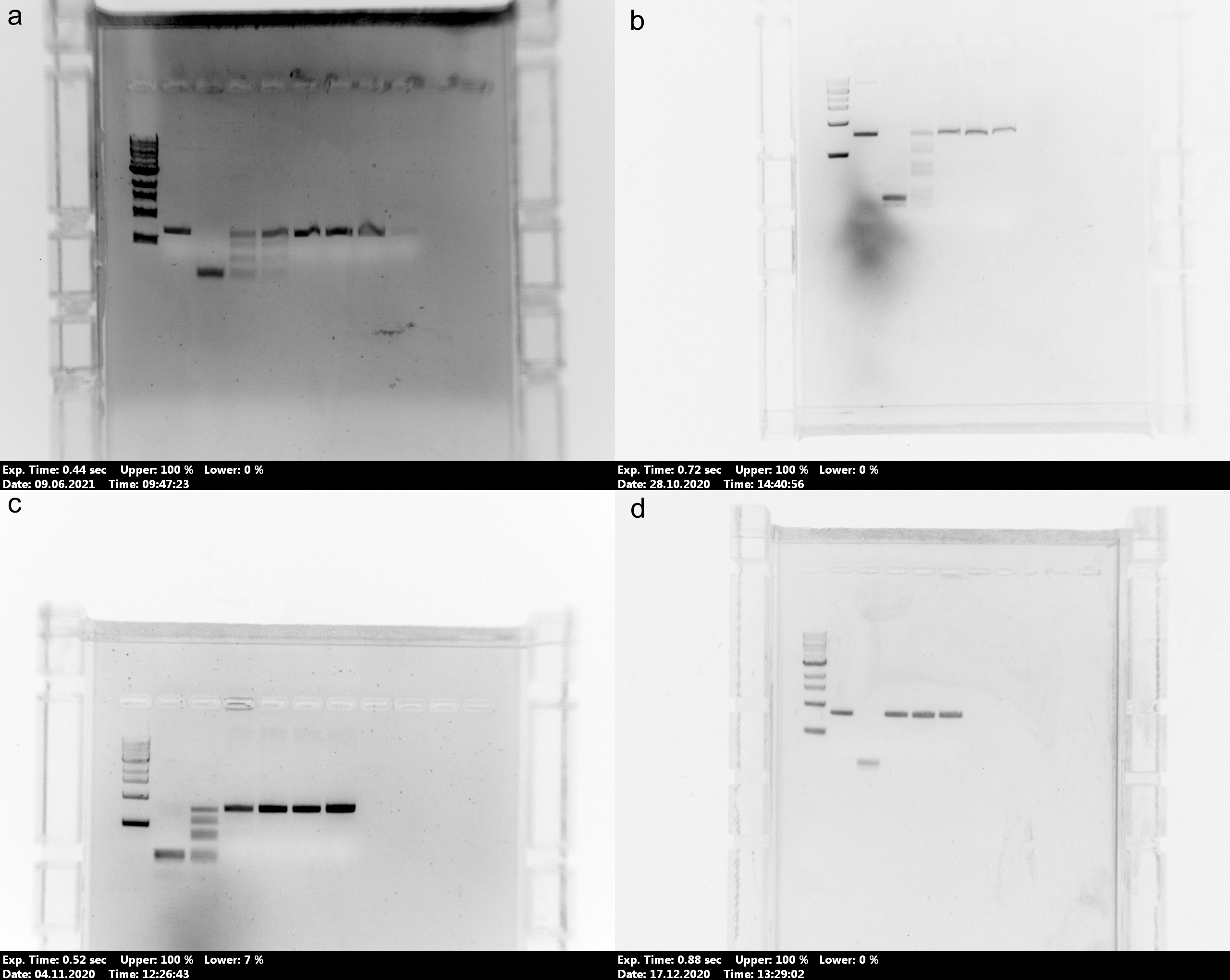

Supplement: Supplementary file 7 — Uncropped EMSAs for Supplementary Fig. 1: a, 4×177; b, 4×187; c, 4×197; d, 4×207 [file 41594_2022_768_MOESM7_ESM.jpg]

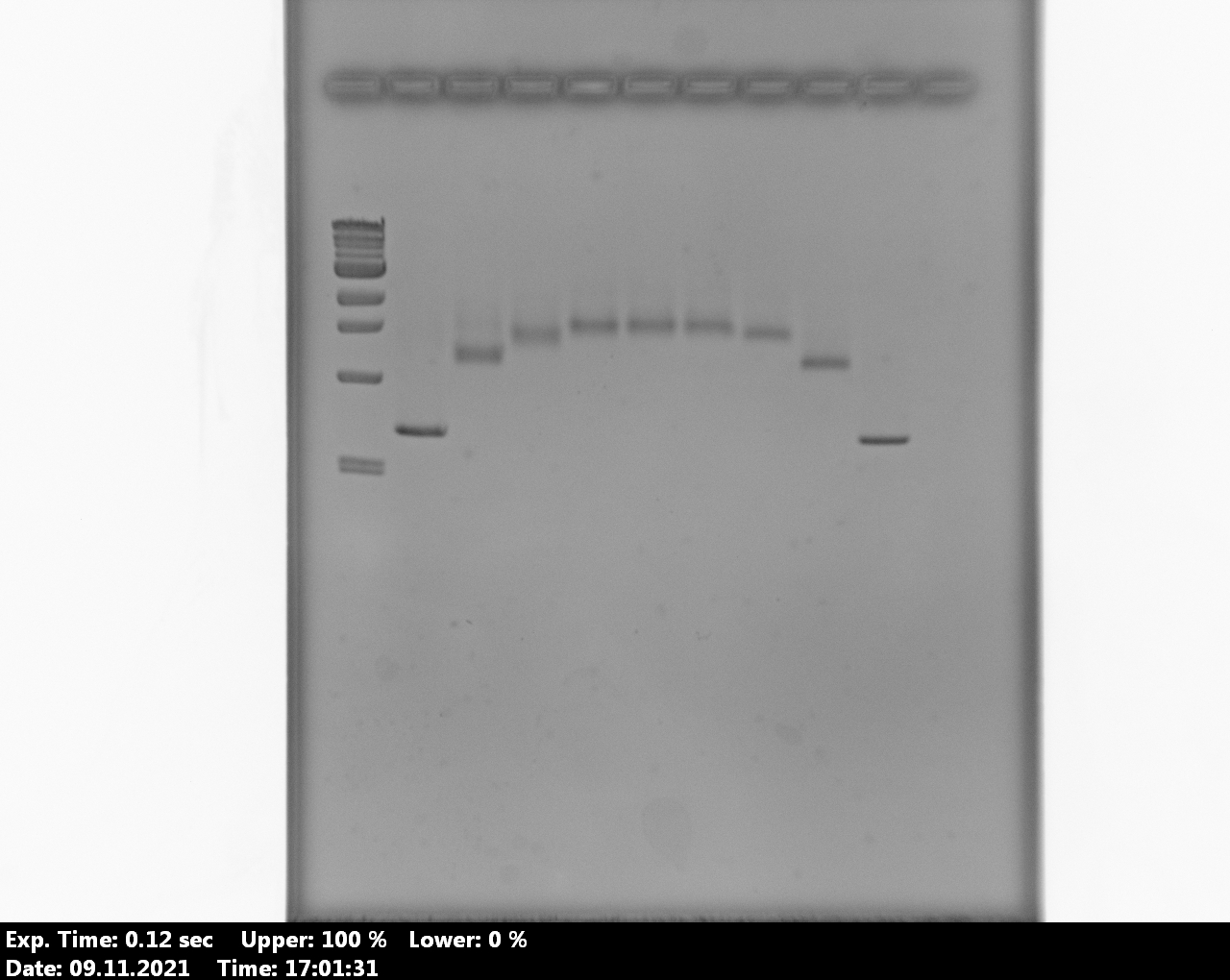

Supplement: Supplementary file 8 — Uncropped EMSA for Supplementary Fig. 11 [file 41594_2022_768_MOESM8_ESM.jpg]

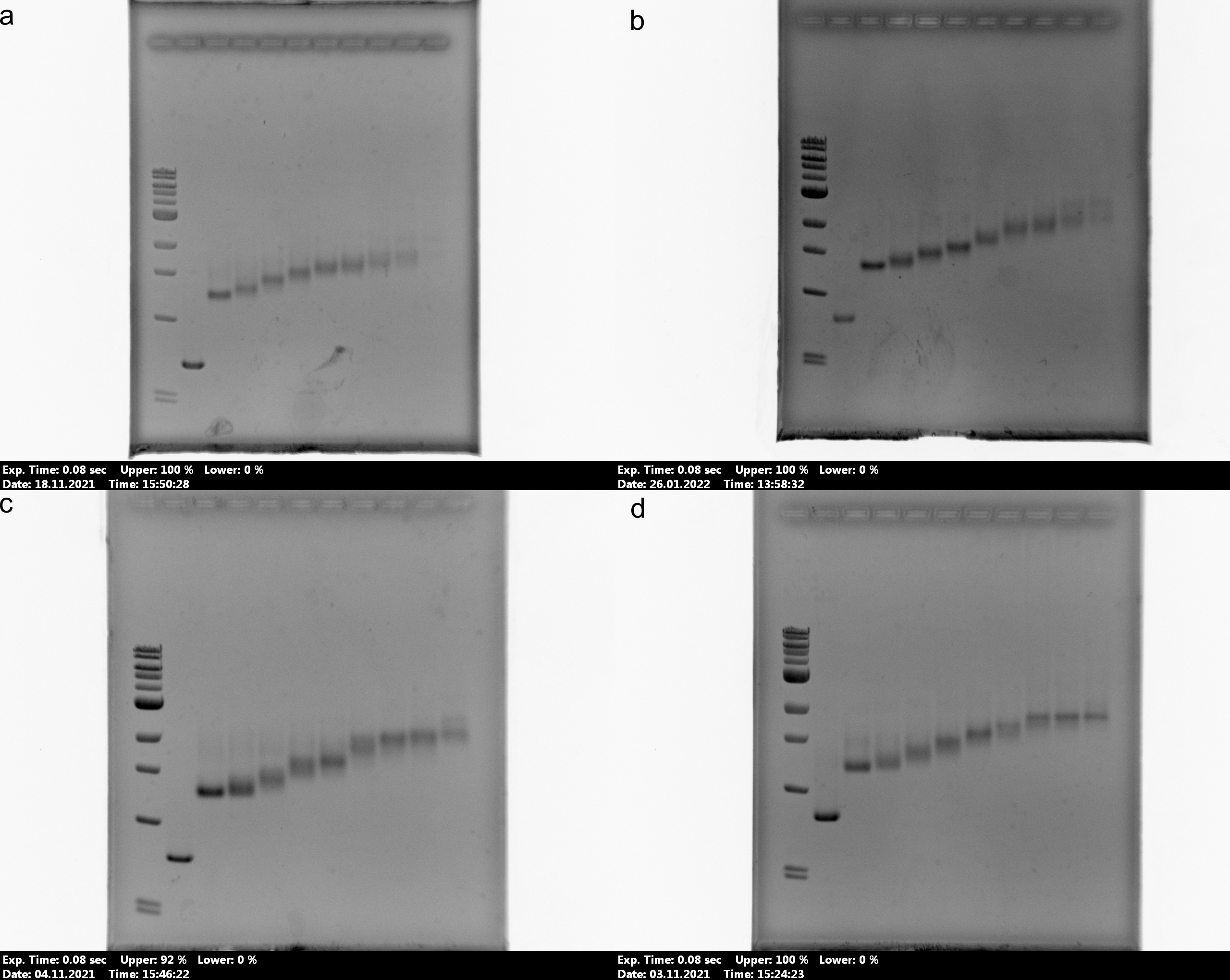

Supplement: Supplementary file 9 — Uncropped EMSAs Supplementary Fig. 12: a, 4×177; b, 4×187; c, 4×197; d, 4×207 [file 41594_2022_768_MOESM9_ESM.jpg]

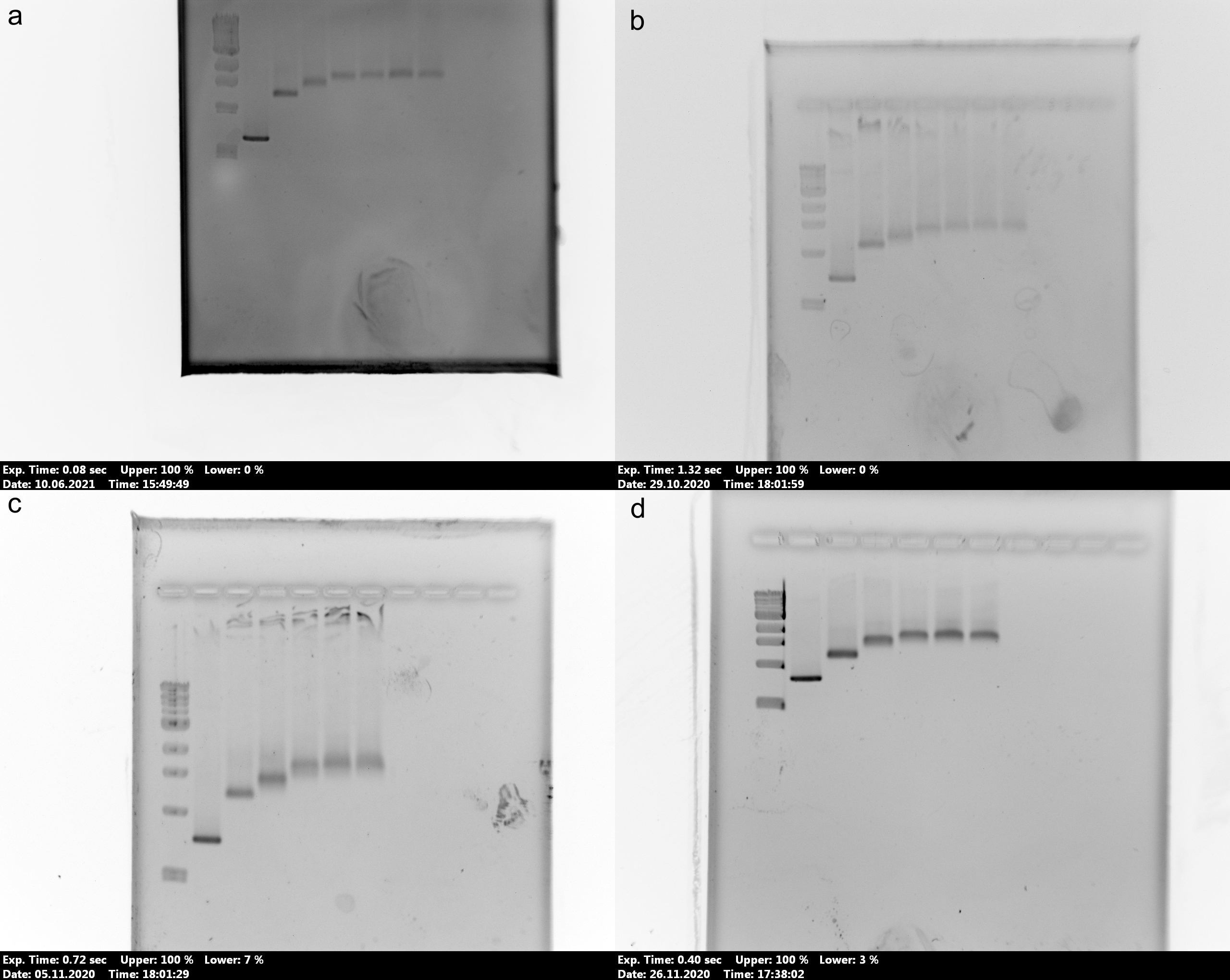

Supplement: Source Data Fig. 1 — Uncropped EMSAs: a, 4×177; b, 4×187; c, 4×197; d, 4×207 [file 41594_2022_768_MOESM10_ESM.jpg]
